# jULIEs: nanostructured polytrodes for low traumatic extracellular recordings and stimulation in the mammalian brain

**DOI:** 10.1088/1741-2552/ac514f

**Published:** 2022-02-28

**Authors:** Romeo R Racz, Mihaly Kollo, Gabriella Racz, Ciprian Bulz, Tobias Ackels, Tom Warner, William Wray, Nikolai Kiskin, Chi Chen, Zhiwen Ye, Livia de Hoz, Ede Rancz, Andreas T Schaefer

**Affiliations:** 1 Sensory Circuits and Neurotechnology Laboratory, The Francis Crick Institute, London, United Kingdom; 2 Department of Neuroscience, Physiology & Pharmacology, University College, London, United Kingdom; 3 Behavioral Neurophysiology, Max-Planck-Institute for Medical Research, Heidelberg, Germany; 4 Department of Anatomy and Cell Biology, Faculty of Medicine, University of Heidelberg, Heidelberg, Germany; 5 Cortical Circuits Laboratory, The Francis Crick Institute, London, United Kingdom; 6 Department of Neurogenetics, Max-Planck-Institute für Experimentelle Medizin, Göttingen, Germany; 7 Sensory gating and subcortico-cortical interactions, Neuroscience Research Center, Charité—Universitätsmedizin, Berlin, Germany; 8 Centre for Electronics Frontiers, School of Electronics and Computer Science, University of Southampton, Southampton, United Kingdom; 9 Department of Bioengineering, Imperial College London, London, United Kingdom; 10 Department of Biological Structure, University of Washington, Seattle, WA, United States of America; 11 The Cortical Circuits, Mediterranean Institute of Neurobiology, Aix-Marseille University, Marseille, France

**Keywords:** extracellular, minimally perturbing, nanostructured, polytrodes, scalable, *in vivo*

## Abstract

**Objective:**

Extracellular microelectrode techniques are the most widely used approach to interrogate neuronal populations. However, regardless of the manufacturing method used, damage to the vasculature and circuit function during probe insertion remains a concern. This issue can be mitigated by minimising the footprint of the probe used. Reducing the size of probes typically requires either a reduction in the number of channels present in the probe, or a reduction in the individual channel area. Both lead to less effective coupling between the probe and extracellular signals of interest.

**Approach:**

Here, we show that continuously drawn SiO_2_-insulated ultra-microelectrode fibres offer an attractive substrate to address these challenges. Individual fibres can be fabricated to >10 m continuous stretches and a selection of diameters below 30 *µ*m with low resistance (<100 Ω mm^−1^) continuously conductive metal core of <10 *µ*m and atomically flat smooth shank surfaces. To optimize the properties of the miniaturised electrode-tissue interface, we electrodeposit rough Au structures followed by ~20 nm IrOx film resulting in the reduction of the interfacial impedance to <500 kΩ at 1 kHz.

**Main results:**

We demonstrate that these ultra-low impedance electrodes can record and stimulate both single and multi-unit activity with minimal tissue disturbance and exceptional signal-to-noise ratio in both superficial (~40 *µ*m) and deep (~6 mm) structures of the mouse brain. Further, we show that sensor modifications are stable and probe manufacturing is reproducible.

**Significance:**

Minimally perturbing bidirectional neural interfacing can reveal circuit function in the mammalian brain *in vivo*.

## 1 Introduction

Systems neuroscience aims to elucidate the causal relationships between an individual’s behaviour and the activity and interaction of neurons and neuronal populations. Correlated activity between mammalian neurons occurs at the millisecond and sub-millisecond timescales. Meaningful features of the sensory input and behaviour have been directly linked to coordinated neuronal activity in various brain areas. The ability to interface with high spatiotemporal resolution in head-fixed and freely moving animals made extracellular electrodes the most powerful instruments to study brain function *in vivo*.

Various electrode materials and manufacturing methods have been developed by the community for penetrating recording probes [1, 2], and have seen wide adoption due to their reproducible manufacturing and relative ease of use. Si-probes [3–5] offer the highest channel densities to date on a single shank. However, the customisation of the sensor layout in three dimensions is limited by the high-overhead fabrication process and potential vascular damage. The total tissue volume displacement could further limit the number of simultaneously implantable probe shanks. Carbon fibre arrays [6–9] are a promising and intensively researched avenue for scalable interfacing. While probe assembly and control of separation between individual fibres is challenging, they allow good quality recordings on a small footprint. Twisted platinum [10, 11] and tungsten wires [12] had been a standard for multi-unit recordings for a long time. While, due to their relatively uncontrolled tip shape and size, local tissue damage is generally significant, the configuration of adjacent placed sensors provides adequate resolution for separating single neuronal unit activity. Polymer fibre polytrodes [13, 14] are especially attractive candidates for long-term recordings. Their mechanical compatibility can potentially alleviate the long-term degradation of signals resulting from brain movements.

Despite these advancements, the interaction of engineered devices with neural tissue remains a key limiting factor in scaling up electrode arrays. Both local tissue damage and long-term deterioration [15, 16] have been found to closely correlate with electrode diameter in various probes, such as microfabricated Michigan and Utah-style arrays [17], MEMS [18], soft polymer or tungsten electrodes [19] and Parylene C probes [20]. Fibre ultramicroelectrodes, electrodes with *µ*m-scale features, are thus highly desirable as their small footprint limits tissue damage. Fabrication, however, poses significant engineering challenges: electrical coupling between the electrode and the bioelectric signals needs to be sufficiently strong despite the small electrode surface area, and concomitantly losses due to stray capacitance need to be minimized.

To overcome these challenges, we designed ultra-low impedance electrodes (jULIEs), a scalable technique for achieving high signal-to-noise electrical recordings and stimulation with fibre ultramicroelectrodes. jULIEs are metal-glass composite microwires drawn to outer diameters (OD) of <25 *µ*m, with metal core diameters (ID) of as little as 1 *µ*m. We introduce a two-step electrochemical modification strategy that reduces electrode coupling impedances by two orders of magnitude. Such modifications enabled high signal-to-noise neural recordings from different brain regions *in vivo*.

Histological and imaging experiments indicated that local vascular damage is minimal. Spikes reached amplitudes of more than 1 mV *in vivo*, indicating that recordings are possible near intact neurons. The layout of recording sites could be arranged in arbitrary patterns tailored to fit neuroanatomical structures and enable laterally dense recordings. Due to these properties, and the scalability of every step in the protocol described here, these electrodes are also very well suited to be used in large-scale, modular recording systems using flat CMOS chips as a recording array (e.g. as described in [21]).

## 2 Materials and methods

### 2.1 Animal welfare

All experiments were performed according to United Kingdom Home Office regulations (Animal, Scientific Procedures Act 1986) or the guidelines of the German animal welfare law and were approved by the local welfare committees and veterinarians.

### 2.2 Ultramicroelectrode fabrication and preparation

**Figure 1:**
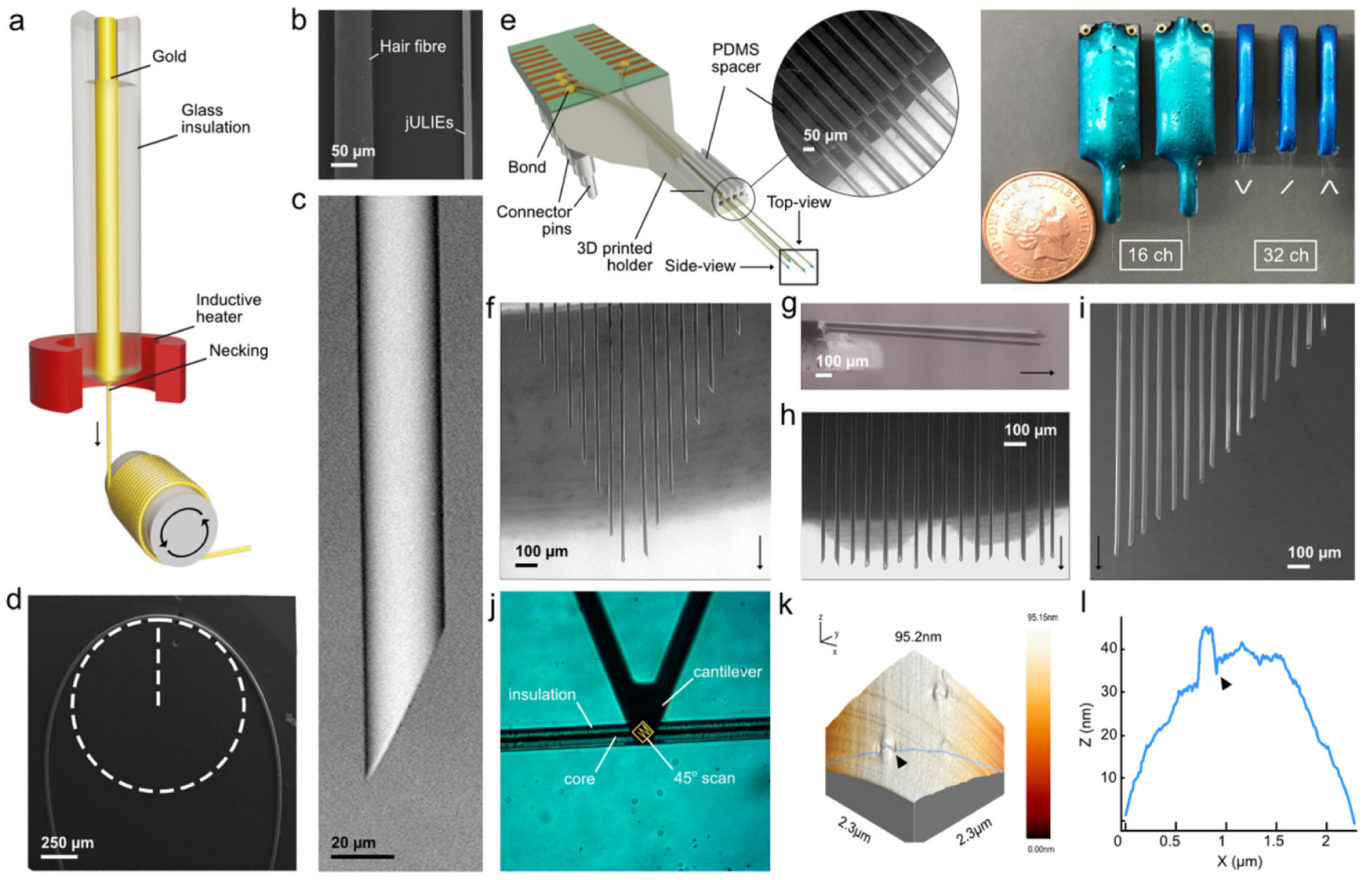
Mechanical properties of the glass-insulated microwires. (a) Apparatus and principle of glass-insulated ultramicroelectrode fabrication by high-speed dieless drawing. A preform containing the intended core metal and glass insulation is inductively heated and drop formed at the necking is continuously spooled up on a drum. (b) SEM image comparing diameters of a human hair and a jULIE fibre. (c) Side-view SEM of a sharpened wire before electrochemical preparation. (d) SEM image of the bending radius of a single fibre. (e) 3D CAD model and assembled jULIEs with a custom layout of 16 and 32 (top-right) and defined fibre separation (inset) (f)–(i) Top-view SEM micrographs of customized probe sensor layouts featuring different geometries with laterally dense packing at 50 *µ*m inter-shank distance. (j) Configuration of the AFM analysis of a randomly chosen portion on the microwire insulation surface. The scan angle is at 45° along the *y*-axis. (k) Arrows highlight a rare extrusion defect of ~8 nm height over a width of ~ 200 nm. (l) AFM cross-section height profile (under blue line) of target plane showing the atomically flat insulator surface. Note the difference in scale between the *x*-axis (*µ*m) and *y*-axis (nm).

**Figure 2:**
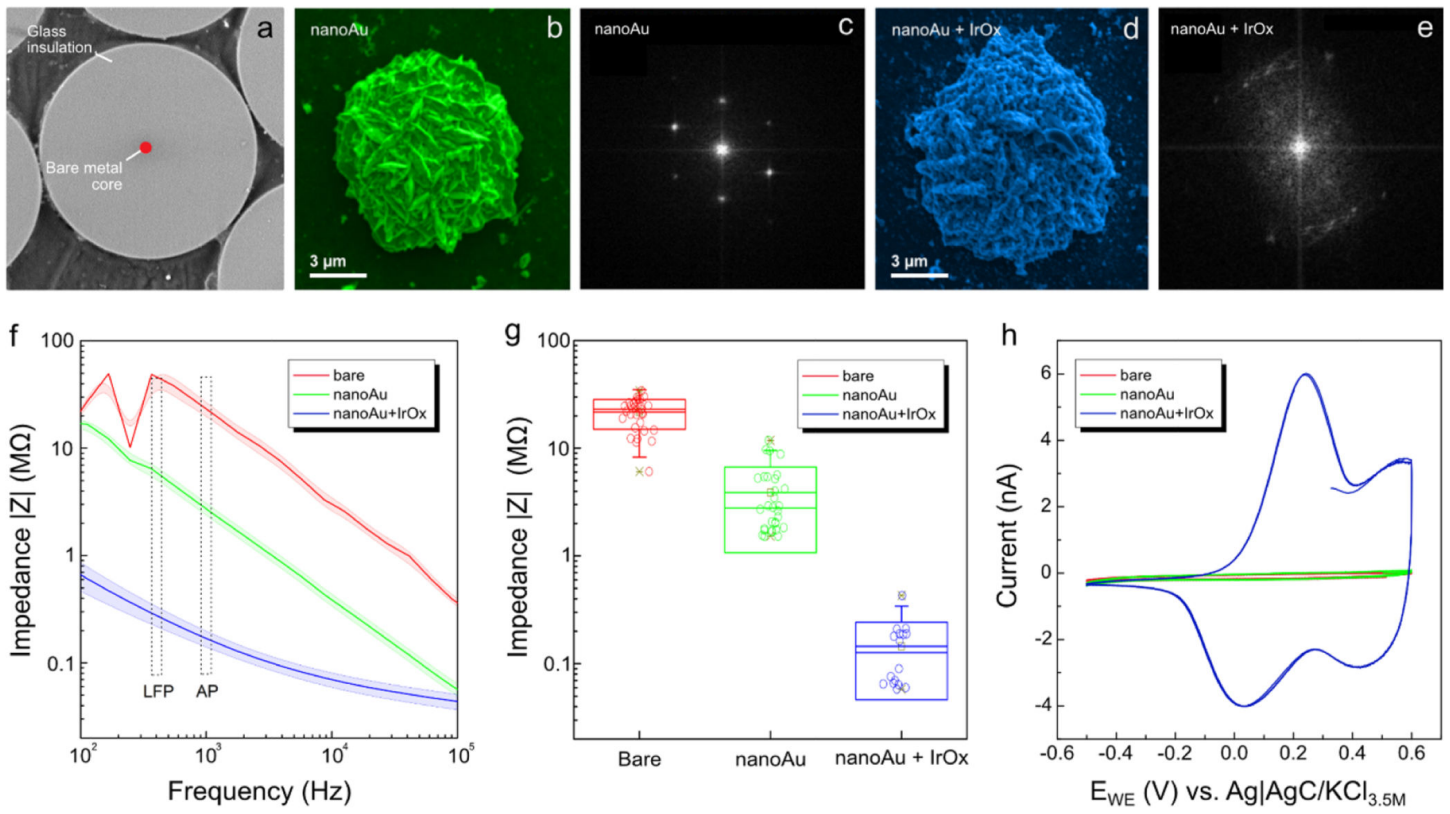
Electrochemical modification of the electrode-electrolyte interface (a) Front-view SEM of flat polished bare fibre. (b) Modification with nanoAu electrodeposited over 35 s. The single-crystal nature of the hemispherical Au deposit is visible in the FFT x-ray diffraction pattern (c). (d) Additional deposition of IrOx on NanoAu. The FFT x-ray diffraction of IrOx shows a typical polycrystalline pattern. EIS of surface preparation stages: (f) Impedance |*Z*| Bode-plot between 100 Hz and 100 kHz. Boxed regions indicate regions of interest for the recording of local field potentials (LFP) or action potentials (APs). Data were collected and averaged from 15 channels per-interface state. Hashed regions represent the standard error of the mean (g) Impedance at 1 kHz sensor state averaged from 31 points for bare, 30 points for nanoAu, and 16 for nanoAu + IrOx (h) cyclic voltammetry response at 50 mVs^−1^ sweep rate of the different sensor preparation stages for an example fibre.

Glass-metal composite ultramicroelectrodes were fabricated using an adapted Taylor-Ulitovsky thermal drawing method [22]. Typically, a cylindrical borosilicate glass tube (OD 10 mm, ID 6 mm, Pyrex, Corning, UK) was loaded with a metal rod (Puratronic, Alfa Aesar, UK) under an inert atmosphere and inductively heated (PowerCube 900, CEIA, Italy) to 850 °C–1000 °C until a separating drop formed as depicted in figure 1(a). The initial thermoformed part was removed, and the flowing fibre was spooled up on a drum at a rate of 2 km min^−1^. Depending on drum speed, drawing temperature, glass composition and preform feeding speed, microwire dimensions could be tailored to core diameters between ~1 and 10 *µ*m with glass insulation thickness up to 15 *µ*m. We worked with the manufacturers (ELIRI) to produce continuously conductive wires with <30 *µ*m in diameter. While we have not verified these numbers for every individual jULIE probe, we have taken SEM images (e.g. like in figures 2(a) and 7(f)) to confirm diameters. Obtained ultramicroelectrodes were bundled together using an Optima 1100 (Synthesis, India) winding machine and embedded in Crystalbond 509 (Agar, USA) a dissolvable thermoplastic resin to fit custom 3D-printed polishing holders. jULIEs were sharpened at 30 degrees using a MetaServ 250 polisher equipped with a Vector head (Buehler, USA) and sequentially polished in five steps using particles (25, 9, 3, 1, 0.05 *µ*m) suspended in water-based emulsion (Buehler, USA) for 40 s each. Sharpened wires were de-embedded by solubilization (1000:1 solid/liquid ratio) in Crystalbond 509 organic stripper for 24 h then washed with isopropanol and dried at 60 °C overnight before long-term storage.

To record *in vivo* extracellular signals, we assembled jULIEs (figures 1(e)–(i), supplementary figure 1 available online at stacks.iop.org/JNE/19/016041/mmedia) into modules of 16, 32 and 128 channels onto custom-designed printed circuit board (PCB) (E44, LPKF Protomat, UK) by a modified instrument and bonding procedure (F&S Bondtec, UK) which allowed *in-situ* read-out of connectivity and was equipped to remove glass insulation. Bonded and sharpened wires were then modified electrochemically as described below.

### 2.3 Electrochemical modifications with nanoAu and IrOx

NanoAu was electrodeposited from a two-part aqueous cyanide bath containing 50 gl^−1^ potassium dicyanoaureate(I) (K_2_[Au(CN)_2_]) and 500 gl^−1^ KH_2_PO_4_ dissolved sequentially in ultrapure deionized water (18 MΩ cm) (Tech, UK) at 60 °C. All reagents were supplied by Sigma-Aldrich, UK and used without further purification. Before electrode-position the probes were washed with deionized water, rinsed with ethanol (90%), wiped with a lint-free cloth (Kimwipes, Kimtech, UK) and dried at 50 °C for 1 h in an oven (Memmert, Germany). The electrodeposition protocol was carried out using a multichannel potentiostat-galvanostat (VSP 300, Bio-Logic, France) equipped with a frequency response analyzer and ultra-low current electrometer controlled with EC-Lab software (Bio-Logic, France). A three-electrode cell setup was composed of the assembled probe acting as working electrodes (*W*_E_), a coiled 1 mm thick platinum wire (PT005150, 99.95%, Goodfellow, US) as a counter electrode (*C*_E_) and a Ag/AgCl|KCl_3.5M_ reference electrode (REF) supplied by BASi, USA (E vs. normal hydrogen electrode = 0.205 V). The REF was kept separated from the bath by a glass tube containing the supporting electrolyte and a porous Vycor glass separator. For nanoAu deposition, the *W*_E_ potential was kept at *E*_red_ = −1.1 vs REF for 35 s. The electrodeposition bath was maintained at 60 °C using a thermostat under vigorous (500 rpm) stirring.

The IrOx electrodeposition was carried out from a modified electrolyte solution based on reported formulations [23, 24] containing 10 gl^−1^ iridium (IV) chloride hydrate (99.9%, trace metal basis, Sigma-Aldrich, Germany), 25.3 gl^−1^ oxalic acid dihydrate (reagent grade, Sigma-Aldrich, Germany), 13.32 gl^−1^ potassium carbonate (99.0%, BioXtra, Sigma-Aldrich, Germany). Reagents were added sequentially to 50% of the solvent’s volume firstly by dissolving IrCl_4_ in oxalic acid followed by the addition of K_2_CO_3_ over a 16 h period until pH = 12 was reached. The electrolyte was aged approximately 20 d at room temperature in normal light conditions until the solution reached dark blue colour as described in supplementary figure 8. The deposition protocol was composed of two consecutive stages combining cyclic voltammetry (CV) and a pulsed potentiostatic protocol (PP). Between protocols, the *W*_E_ was kept at the open-circuit voltage (OCV) for 180 s to allow equilibration. During CV deposition the W_E_ potential was cycled 50 times between −0.50 and 0.60 V vs REF at 1 Vs^−1^ in both anodic and cathodic directions (supplementary figure 3(a)). During the pulsed potentiostatic deposition the W_E_ potential was stepped 500 times between 0 to 0.60 V vs REF in 1 s steps (supplementary figure 3(b)). nanoAu electrodeposition baths were freshly made while IrOx baths were sampled from a larger batch before each deposition run.

### 2.4 jULIEs electrochemical characterization

Electrochemical impedance spectroscopy (EIS) and CV was used to characterize the sensor sites individually in: the unmodified state (bare surface), after modification with nanoAu, and nanoAu + IrOx respectively. Multichannel jULIE modules were assembled and connected to the potentiostat using custom-designed PCBs and matching connector plugs. Characterization was done in 150 mM phosphate-buffered saline (PBS) using a 3-electrode (see details for *C*_E_ and REF above) for their CV response and EIS profiles. EIS measurements were performed by applying a 10 mV sinewave around the OCV in the frequency range 1 to 100 kHz with three consecutive measurements per frequency point and five repetitions for each channel. Using an identical cell setup, CV response was recorded for individual channels by sweeping electrode potential from −0.5 and 0.6 V vs REF with 100 mVs^−1^ sweeping rate in both cathodic and anodic directions. EIS and CV were performed using the same instrumentation and setup described in section 2.3.

### 2.5 Morphological, compositional, and structural characterization

Modified sensor sites were characterized by their microstructure, atomic lattices and chemical composition by field emission scanning electron microscopy and scanning transmission electron microscopy (STEM) using a multipurpose 200 kV JEOL JEM-2100F transmission electron microscopy (TEM) analytical electron microscope coupled with an energy dispersive x-ray spectrometer (EDS) and Oxford Instruments INCA/Aztech EDS 80 mm X-Max detector system for elemental analysis with nanometre spatial resolution. A dual-beam FEI Helios 600 FIB SEM^−1^ system equipped with a gallium ion source operating in the accelerating voltage range 0.5–30 kV and an Omniprobe (FEI Helios) micromanipulator was used to morphologically characterize and prepare samples for TEM imaging. Sample preparation consisted of: (a) deposition of a protective platinum (Pt) layer by sputtering onto the specimens, (b) milling a thin slice perpendicularly to the sample surface, (c) extraction and glueing the specimen slice to a TEM grid, and (d) further thinning of the sample with low-voltage focused ion-beams at grazing incidence until an electron-transparent region was obtained. These steps were repeated for both nanoAu and nanoAu + IrOx specimens as depicted in supplementary figures 2 and 3.

### 2.6 Atomic force microscopy measurements

The surface of the microwire in contact with the tissue was measured by atomic force microscopy (AFM) using a Dimension FastScan Bio AFM (Bruker, Santa Barbara, USA). Individual fibres were rinsed in ethanol, dried in air and fixed to glass plates with Kapton-tape at both ends. AFM imaging was carried out in contact mode using silicon nitride probes with a force constant of 1.5 N m^−1^ and a resonant frequency of 13 kHz. The direction of the scan was 45° to the axis of the wire as it provided the best SnR for the current setup. The 3D profiles for figure 1(l) were collected using custom software from Horcas and Fernandez [25].

### 2.7 Local field potential modelling

For simulation of the LFP around a detailed mitral cell model, we used a Neurolucida reconstruction of a mitral cell (IF04208 from [26]). Ion channel densities for different domains (glomerular tuft, apical dendrite, lateral dendrite, soma, axon) were adapted from Rubin, 2006 [27]. To gain a more accurate picture of the field around the initial segment, a sodium channel density of 2000 pS *µ*m^−2^ [28, 29] was included in the first 5 *µ*m of the axon. The LFP was simulated at different locations with the line-source method [30] using the LFPy Python package [31].

### 2.8 Histology

Tissue integrity post jULIE insertion was determined by histological methods as described [21]. In brief, after craniotomy, 0.2 ml of 0.5% Evans Blue was injected into the tail vein. Before insertion, jULIEs were dipped into SP-DiO (Molecular Probes, OR, USA) and allowed to dry. After the jULIEs were removed from the brain the mouse was perfused with icecold 4% PFA, the brain was harvested and stored in 4% PFA overnight. Using a Vibratome (Leica, Germany), the brain was sliced into 100 *µ*m horizontal sections. Slices were stained with DAPI using a 1:1000 DAPI:PBS wash for 10 min, transferred to fresh PBS, mounted and sealed. Imaging was completed on a confocal microscope (Leica SP5). Control silicon probe insertions were performed with a NeuroNexus A1x16-3 mm-25-177 probe (15 *µ*m thickness, width between 50 and 77 *µ*m). The same insertion speed was used for both jULIE and Si probes.

### 2.9 Extracellular recordings in the olfactory bulb

To test jULIEs, we performed recordings *in vivo* in the OB of mice. 4–6 weeks old C57BL/6 mice were 4 anaesthetized using a mixture of ketamine (100 mg per kg of body weight) and xylazine (20 mg per kg for induction and 10 mg per kg for maintenance) administered intraperitoneally and supplemented as required. Body temperature was maintained at 37 °C using a feedback-regulated heating pad (FST, USA). The main olfactory bulb (MOB) was accessed through a 2 × 2 mm craniotomy window after fixation of the skull with a head-plate. The surface of the brain was protected by an imaging well containing Ringer’s solution and a caudally fixed Ag|AgCl reference electrode. Probes with site impedances ranging between 300 kΩ to 1 MΩ were lowered to a depth of approximately 400 *µ*m using micromanipulators (Luigs & Neumann, Germany). Extracellular recordings were performed using a Tucker Davis RZ2 amplifier with a RA16AC-Z head-stage or an Intan RHD2132 headstage on an OpenEphys amplifier (openephys.org). Mice were presented with mixtures of odorants (Mixture 1: Ethyl butyrate & 2-hexanone, Mixture 2: Eucalyptol & Amamyl acetate) (Sigma Aldrich, USA) diluted 1:5 with mineral oil. Units were isolated using either Spike2 (Cambridge Electronics Devices, Cambridge, UK) or Kilosort [32] and units with well-defined auto-correlograms were selected for further analysis. Units were found with distinct responses to different odours, that were stable across repeats.

### 2.10 Extracellular recordings in the inferior colliculus

Female C57BL/6JOlaHsd (Janvier, France) mice were anaesthetized with Avertin (0.15 ml/10 g). Additional doses (0.03 ml/10 g) were given as needed to maintain anaesthesia. After anaesthesia, the animal was fixed with blunt ear bars on a stereotaxic apparatus (Kopf, Germany). Body temperature was maintained at 36 °C with a feedback-regulated heating pad (ATC 1000, WPI, Germany). Vidisic eye gel (Bausch + Lomb GmbH, Germany) was used to prevent the eyes from drying out. A metal head-holder was glued to the skull 1.0 mm rostral to Lambda with methyl methacrylate resin (Unifast TRAD, GC). A craniotomy of 0.8 × 1.0 mm with the centre 0.85 mm from the midline and 0.75 caudal to Lambda was made to expose the left inferior colliculus (IC). Dura was carefully removed, and the surface of the brain was protected with Saline (B. Braun, Germany). The IC was identified by its position posterior to the transverse sinus and anterior to the sigmoid sinus. With a micromanipulator (Kopf, Germany), jULIEs with impedances between 300 kΩ and 1 MΩ were lowered vertically and advanced into the IC. The electrophysiological signal was amplified (HS-18 MM, Neuralynx, USA), sent to an acquisition board (Digital Lynx 4SX, Neuralynx, USA), and recorded with a Cheetah 32 Channel System (Neuralynx, USA). The voltage traces were acquired at a 32 kHz sampling rate with a wide band-pass filter (0.1–9 kHz).

The sound was synthesized using MATLAB, produced by a USB interface (Octa capture, Roland, USA), amplified (Portable Ultrasonic Power Amplifier, Avisoft, Germany), and played with a free-field ultrasonic speaker (Ultrasonic Dynamic Speaker Vifa, Avisoft, Germany). The speaker was positioned 15 cm away from the right ear. The sound intensity was calibrated with a Bruël & Kjaer microphone. For measuring the tonal receptive field, we used sound stimuli consisting of 30 ms pure tone pips with a 5 ms rise/fall slope repeated at a rate of 2 Hz. Thirty-two frequencies (2 kHz to 47 kHz, 0.16-octave spacing) were played in a pseudorandom order at intensities of 60 or 70 dB. Each tone-intensity combination was played five times.

The recorded voltage signals were high pass filtered at 500 Hz. The root-mean-square (RMS) of the noise of each channel was calculated as the RMS level of the filtered trace during the first 10 s of recordings, which included spontaneous and evoked activity. To improve the signal-to-noise ratio of the recording, the common average reference was calculated from all the functional channels and subtracted from each channel [33]. For multiunit analysis, spikes were detected as local minima below a threshold of six times the median absolute deviation of each channel. If the calculated value was higher than −40 *µ*V, the threshold was set to −40 *µ*V. To analyze the sound-driven responses, peri-stimulus time histograms were built by aligning the signals at stimulus onset and calculating the number of spikes/ms. Iso-intensity tuning curves were built from the sum of spikes in an 80 ms window from stimulus onset, at each intensity as a function of frequency.

### 2.11 Extracellular recordings in the L5 of the primary visual cortex

Male C57BL/6 mice between 2 and 4 months old were used for acute recordings in the visual cortex. For surgery, mice were anaesthetized with isoflurane (3% induction followed by 2% maintenance) and fixed on a stereotaxic apparatus (Model 940, David Kopf Instruments, Germany) using ear bars. Body temperature was maintained at 36 °C with a DC temperature regulation system (FHC, Inc. USA). A skin incision was made, and the exposed skull was cleaned and dried. A metal headplate was cemented to the skull using dental cement (Super-Bond C&B, Sun Medical, Japan). Animals were allowed to recover after surgery in their home cage. Analgesia (2 mg kg^−1^ Meloxicam with 0.1 mg kg^−1^ Buprenorphine) was provided. On the day of the recording, a craniotomy 1.5 mm long, 0.3–0.5 mm wide was made with small drill bits on the right hemisphere under isoflurane anaesthesia (3% induction followed by 2% maintenance). The long axis of the craniotomy was parallel with the lambda suture. The dura was removed, and the craniotomy was covered with a silicone-based sealant (Kwik-cast, World Precision Instruments). Following recovery from surgery (4–24 h), the animal was lightly anaesthetized in 1.0%–1.5% isoflurane and head-fixed to the recording setup. The craniotomy was kept moist with cortex buffer (NaCl 125 mM, KCl 5 mM, HEPES 10 mM, MgSO_4_ 2 mM, CaCl_2_ 2 mM, Glucose 10 mM, pH 7.4) and the jULIEs (<200 kΩ at 1 kHz impedance per site) or Si-probe (A4X1-tet-3 mm-150-121, Neuronexus, USA) was slowly (~10–20 *µ*m min^−1^) inserted into primary visual cortex using a micromanipulator (SM1, Luigs & Neumann, Germany). Neural signals were recorded using a PZ2-32 preamplifier and an RZ2 BioAmp Processor (Tucker-Davis Technologies, USA). Data were acquired at a 24.4 kHz sampling rate and recorded using TDT’s OpenEx software suite. The amplifier ground was connected to a screw implanted in the skull.

For visual stimulation full screen drifting gratings of eight different directions (spatial frequency: 0.08 cpd; temporal frequency 2 Hz; 2 s long, with 2 s grey screen in between), were presented in randomized order on a 27 inch LCD screen (E2711T, LG Electronics), placed 15-25 cm from the left eye. Gratings were generated and presented using MATLAB (Mathworks, USA) and the Psychophysics toolbox [34].

Data were extracted and processed using custom-written Matlab scripts. Neural signals were bandpass filtered at 300-5000 Hz for spike detection. Semi-automatic spike sorting was carried out using Klusta software with default parameters [35]. Clusters with a clear refractory period in the auto-correlogram (ISI violation <0.5%) and isolation distance >20 were classified as single units. Manual curation of resultant clusters was done using Phy [35].

### 2.12 Extracellular recordings of deep brain areas

The 4-6 week old C57BL/6 mice were anaesthetised as described above with olfactory bulb recordings. A craniotomy roughly 2 × 2 mm was made equidistant between the bregma and lambda, as close to the sagittal suture as possible. A well was constructed from silicon (Kwik-cast, World Precision Instruments) around the craniotomy and was filled with cortex buffer (NaCl 125 mM, KCl 5 mM, HEPES 10 mM, MgSO_4_ 2 mM, CaCl_2_ 2 mM, Glucose 10 mM, pH 7.4). The dura over the craniotomy was carefully pulled back using a bent 29 G needle. The probe was positioned over the craniotomy and zeroed when the frontmost wire was seen to touch the brain surface. An Ag/AgCl reference electrode was immersed into the well. The probe was advanced at no faster than 5 m s^−1^ until spiking activity was detected across most channels. The probe was stationary at each depth for between 2 and 5 min. The probe was then advanced again at no faster than 5 *µ*m s^−1^. This advancement and stationary recording were repeated several times until a depth of 6037 *µ*m was reached, at which point the probe was advanced no further.

### 2.13 *In vivo* two-photon imaging of jULIE insertion into the olfactory bulb

C57BL/6 mice were anaesthetized using a mixture of Fentanyl/Midazolam/Medetomidine (0.05/5/0.5 mg kg^−1^). The skull overlying the dorsal olfactory bulb was thinned using a dental drill and removed with forceps, the dura was peeled back using fine forceps. Body temperature was maintained at 37 °C throughout the experiment using a feedback-controlled heating pad (FHC, Inc. USA). Sulforhodamine 101 (Sigma Aldrich, 100 *µ*m final concentration) was injected intraperitoneally to label blood vessels. Animals were then moved to a two-photon microscope (Scientifica Multiphoton Vivo-Scope) coupled with a MaiTai Deep See laser (Spectra Physics, Santa Clara, CA) tuned to 940 nm (~50 mW average power on the sample) for imaging. Images (512 × 512 pixels) were acquired with a resonant scanner at a frame rate of 30 Hz using a 16 × 0.8 NA water-immersion objective (Nikon). For *in vivo* z-stack imaging, images were taken at a resolution of 512 × 512 pixels with 2 *µ*m *z* intervals. Wires were dip-coated in DiO (Sigma Aldrich) before insertion into the dorsal olfactory bulb using a micromanipulator (Scientifica, Uckfield, UK). Images were analysed post hoc using ImageJ (NIH, Bethesda).

### 2.14 *In vivo* two-photon imaging of stimulation in the olfactory bulb

For *in vivo* imaging, we used transgenic mice expressing the genetically encoded Ca^2+^ indicator GCaMP6f in projection neurons as before (Tbet-Cre x Ai95, see Ackels [36]). Mice were prepared for imaging as described in the previous section. Wires were dipcoated in DiI (Sigma Aldrich) before insertion into the dorsal olfactory bulb using a micromanipulator (Scientifica, Uckfield, UK). A silver/silver chloride CE was inserted into caudal parts of the imaging well. For electrical stimulation, typically 100 ms long step current pulses were applied repeatedly to the inserted microwire relative to the caudal reference electrode with a Digitimer NL800A stimulator while imaging at typically six *z*-planes (∂z = 25 *µ*m). The total frame rate was 30 Hz, resulting in an effective volume repetition rate of 5 Hz. Stimulation strength was varied between 5 and 85 *µ*A in 5 *µ*A steps and repeated 3-5 times for each stimulation strength. ROIs were selected manually offline using custom-written routines in ImageJ and data was exported for further analysis in Matlab.

## 3 Results

### 3.1 Electrode fabrication and probe assembly

jULIE neural probes were assembled (figure 1 and supplementary figure 1) from sharpened composite glass-metal microwires. Microwires were mass-produced through a modified die-less Taylor-Ulitovsky (TU) drawing method [22, 37] (figure 1(a)). Overall microwires were small (figure 1(b)), continuously insulated (figure 1(c)) and conductive over several 100 m, yet remained flexible with ~500 *µ*m bending radius (figure 1(d)). TU drawing resulted in a smooth exterior glass surface (figure 1(c)), as measured by AFM (figures 1(j)–(l)), and a well-defined glass-metal boundary (figure 2(a)). Before assembling into arrays (supplementary figure 1) individual wires were prepared as follows: Wires were rewound from the production drums into bundles. These were then embedded in a soluble thermoplastic (see Methods section 2.2) and polished in bulk to approximately 30 degrees (figure 1(c), supplementary figures 1(a)–(c)). When released from the common matrix, this resulted in individually sharpened fibres designed to reach into deep structures [21, 38–40] acutely.

Given the small diameter of the metal cores, the exposed metal surfaces that resulted from sharpening were small (<few 10 *µ*m^2^). Consequently, the exposed metal surfaces had characteristically large impedances |*Z*| in the frequency bands of interest for neural recordings (1 Hz to 100 kHz see figures 2(f) and (g)). This results in signal attenuation and increased noise [41–43] and it is a known challenge for the design of ultra-thin metal electrodes. Electrical coupling to the extracellular signal can, in general, be improved by roughening the electrode surface by physical, chemical, and electrochemical means. However, this often results in uncontrolled modifications and can lead to mechanical failure [15]. Both organic and inorganic materials have been used to reduce electrode impedances albeit for larger sensor surfaces [2]. Iridium oxide (IrOx) electrodeposition for example is a well-established surface modification for extracellular recording electrodes [23, 24, 44].

Thanks to its faradaic nature and stable reversible Ir^3+^/Ir^4+^ redox couple it also provides an excellent substrate for microstimulation [45].

To maintain the cylindrical profile and small footprint of the jULIE tip, our aim was to increase the specific surface area of the metal sensor while keeping the geometrical size increase controlled and minimal. We established a three-step microwire surface modification protocol (figure 2, supplementary figures 1–3). First, after the polishing step described above (supplementary figures 1(a)–(c)) the metal core was exposed as a disk uniformly surrounded by insulation (figure 2(a)). Secondly, under potentiostatic control, lamellar gold nanostructures (nanoAu) were deposited (figures 2(b), (c) and supplementary figure 2) from an additive-free cyanide electrolyte (see Methods). While microwire cores can be produced from a wide range of metals, potential chemical incompatibility with the tissue may occur, causing toxicity or corrosion. The deposition of gold nanostructures decouples the microwire core from the electrolyte, allowing the usage of custom conductive materials, largely independent of their toxicity or corrosion properties. In the third step, nanoAu was further modified with a film of polycrystalline IrOx (figures 2(d), (e) and supplementary figure 3) from an aged IrCl_4_ electrolyte (see Methods). This was performed by a combination of CV and potentiostatic pulsed electrodeposition (PD) protocols. During the first deposition sweeps by CV, nucleation centres developed on the substrate (as shown e.g. for MnO_2_ [46]) and the charge was stored with each voltammetry cycle. Similarly, for IrOx deposition (supplementary figure 3(a)) CV facilitated the development of IrOx on nanoAu and subsequent potentiostatic pulses accelerated build-up (supplementary figure 3(b)).

The resulting films of polycrystalline IrOx (supplementary figures 3(d)–(f)) evenly covered the nanoAu substrate (figure 2(d)). Depending on the dimensions of the nanoAu deposit and IrOx film thickness, in combination, nanoAu + IrOx reduced interfacial impedances by up to two orders of magnitude (figures 2(f)–(h)) to around 100 kΩ at 1 kHz. These values were further adjustable through modification of the number of pulses and voltammetry cycles to match e.g. size constraints or input impedances of amplifiers.

**Figure 3:**
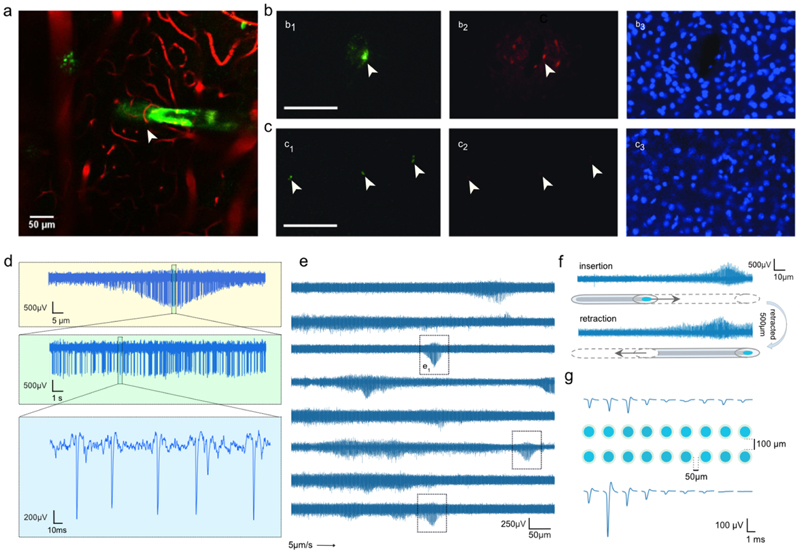
Structural and functional response of tissue at probe insertion. (a) *In vivo* acute 2-photon microscopy observation of insertion of a DiO-labelled jULIE (green) into the MOB with sulforhodamine labelled capillaries (red). jULIEs were moved along its axis towards the middle of the OB then retracted several times (see supplementary video 1). (b) Acute tissue damage upon insertion of a Si-probe (b_1_)–(b_3_) and of a multi-shank jULIE probe (c_1_-c_3_; probe with three jULIEs). Insertion sites are labelled by DiO (b_1_, c_1_; green). Damage to the blood-brain barrier is indicated by the extravasation of albumin-bound Evans Blue (b_2_, c_2_; red), and displacement of cell nuclei (b_3_, c_3_; DAPI, blue). (d) Details of extracellular recording on a single channel during electrode insertion at constant speed ~5 *µ*m s^−1^ near a spontaneously firing unit. (e) Spontaneous multi- and single-unit activity recorded on 8 parallel jULIEs moved from the top of the brain to ~1 mm deep (e_1_-box) typical appearance of single a single unit along the travelling path. Spontaneously active units appeared as humps of increased amplitude on top of the multi-unit activity. (f) Activity was preserved when jULIEs were inserted and retracted multiple times along the same axis; unit activity was recovered in a comparable location during insertion and retraction (despite ~500 *µ*m further insertion of the probe). (g) Single-unit amplitude distribution on a custom arranged rectangular recording site layout featuring 50 *µ*m lateral inter-shank and 100 *µ*m inter-layer distance.

The combination of thermal noise from the rodent cortical surface (average of 34.4 °C) and a typical (50 kΩ) sensor impedance at 30 kHz resulted in 5 *µ*V RMS noise voltage and a noise power of −128.9 dB. Signal-to-noise varied between −10 dB for single units recorded from <20 *µ*m (figures 3(d) and (e)) between the sensor and the cell, and −30 dB for multi-units for an average 200 *µ*V signal.

Electrodeposits were mechanically stable during manipulation, tissue insertion and penetration in deep brain structures (supplementary figure 4, see also figure 8 for stable recordings at depths >6 mm).

To gain more insight into the robustness of the deposits we determined stability by EIS 3-5 mm above the brain surface before insertion, in contact with the brain surface and after acute insertion to 1300 *µ*m. On a 16-channel probe, impedance changed by 200–500 kΩ from before to after insertion (supplementary figure 4 for 1 kHz). These are likely due to resistive changes at the interface as observed by others [47] and did not impact recording performance in acute preparations. Further, we measured impedance over a broad range of frequencies after 1 million stimulation pulses (600mV 5 ms long) at the water-window limit (supplementary figure 6) This resulted in a change from 200 to 800 kΩ, still well within the range of impedances useful for electrical recordings *in vivo*.

For extracellular recordings in mice, 6–8 cm long microwires were assembled into probes composed of a low-profile 3D-printed holder incorporating a custom PCB, a connector plug with matched channel count, and a polydimethylsiloxane (PDMS) spacer to control the inter-fibre distance (figure 1(e) inset, 8 supplementary figure 1). Given the robust mechanical properties of the fibres, they were handled with relative ease using general lab instrumentation. Individual fibres were connected to PCBs by a modified ball-bonding technique shown in supplementary figure 7. In this semi-automatic process, individual wires were packed in various 2D and 3D arrangements with horizontal and vertical spacings varying between 5 to 150 *µ*m between fibres (shown for 50 *µ*m inter-shank separation in figures 1(f)–(i)). Variability was measured through SEM and ranged between 10 and 50 *µ*m for shanks up to ~5 mm long and up to ~250 *µ*m for 10 mm long shanks. This variance originated from the fibres being stored on small radius spools and was found to be less pronounced on fibres with smaller core diameters. The risk of misalignment was further mitigated by unspooling the fibres well in advance. The overall sensor site layout was tailored to fit the neuroanatomical structure of interest, i.e. 1× row of equidistant sensors for the mitral-cell layer in the olfactory bulb of mice. Overall, the multi-shank probe fabrication, from the component assembly, sensor modification, and characterization for a standard 16 channel probe was approximately 150 min, comparable with tetrode fabrication.

### 3.2 Tissue insertion

To validate the technology, we inserted jULIEs into the olfactory bulb (OB) of 4-6 week-old anaesthetized mice (figures 3(a)–(g)). In standard recordings using Si-probes, unit activity was typically suppressed, up to ~45 min after insertion at 1 mm s^−1^ as described by Fiáth *et al* [48]. In contrast, both single and multi-unit activity could be reliably resolved immediately after jULIE insertion starting from superficial layers (figures 3(e) and 6(d)), suggesting that tissue integrity was only minimally perturbed.

To directly assess the tissue, we monitored the integrity of the microvasculature. We coated jULIEs with DiO, labelled the blood vessels with sulforhodamine, and imaged tissue structure during insertion using 2-photon microscopy. While individual fibres were stiff enough to penetrate, they were sufficiently compliant to preserve the structure. Capillaries folded around the fibre shank and slid on its surface without dragging the surrounding tissue or causing apparent rupture to the blood vessels (figure 3(a) and supplementary video 1). To assess the latter more directly, we performed histology after insertion experiments, where the vasculature was loaded with Evans-Blue (figures 3(b) and (c)), a dye unable to penetrate the blood-brain barrier due to its high-affinity binding to albumin [49]. For standard silicon probes (figure 3(b)) insertion resulted in significant local damage to the blood-brain-barrier as indicated by extravasation of Evans Blue (figure 3(b_2_)) and tissue displacement with loss of neuron density around the rectangular shank (figure 3(b_3_)). Insertion of the jULIEs, on the other hand (figure 3(c)), left cell density seemingly unperturbed (figure 3(c_3_)) and resulted in no detectable damage to the blood-brain barrier (figure 3(c_2_)).

### 3.3 *In vivo* extracellular recordings

We recorded spontaneous single and multi-unit activity with a high signal-to-noise ratio while slowly inserting jULIEs into the MOB (figure 3, supplementary figure 5). At insertion rates of ~5 *µ*m s^−1^, we readily detected the emergence of unit activity (figures 3(d) and (e)). Action potentials emerged from the background multi-unit levels to a maximum (figure 3(e_1_)), as sensors were advanced by 10–100 *µ*m and subsequently decreased over similar length scales with further insertion. The highest amplitudes exceeded 1 mV suggesting proximity of the recording site to the intact neuron at that point [50, 51].

When jULIEs were inserted and retracted multiple times on the same axis around a spontaneously firing unit, activity was preserved at the same location over several insertion-retraction cycles (shown for the first cycle in figure 3(f)), even when they were inserted beyond the recorded unit by several hundred micrometres. This suggests that the local neural network remained structurally and functionally intact along the insertion track. The electrode neither caused detectable damage to, nor displaced neurons upon insertion as shown in figure 3(f).

Electrical recordings were not performed simultaneously with imaging in this study. Recently, however Cecchetto [52] confirmed the possibility of using the two techniques simultaneously with highly reduced optical noise and no photo-induced interferences in the electrical recordings.

Tetrodes typically have a larger outer diameter, uneven tip and shank geometry (twisted wires are cut with a blade), thus resulting in noticeable insertion tracks through local damage and cell displacement. The tetrode configuration, however, allows recording of units on multiple electrodes, improving and simplifying spike sorting. We thus wanted to assess whether jULIEs arranged in a laterally dense configuration with individual electrodes spaced by 50–100 *µ*m would similarly enable detecting individual units on multiple recording sites. Thus, we constructed a jULIE array formed by two stacked layers of nine probes each with 50 *µ*m inter-shank spacing and 100 *µ*m inter-layer distance. We observed that single active units were indeed resolved on several adjacent channels (figure 3(g)), making it possible to adapt well-established spike-sorting algorithms such as Klustakwik [35], KiloSort [32], Offline Sorter (https://plexon.com/products/offline-sorter/) or MountainSort [53].

### 3.4 Electrode—neuron distance estimation

**Figure 4:**
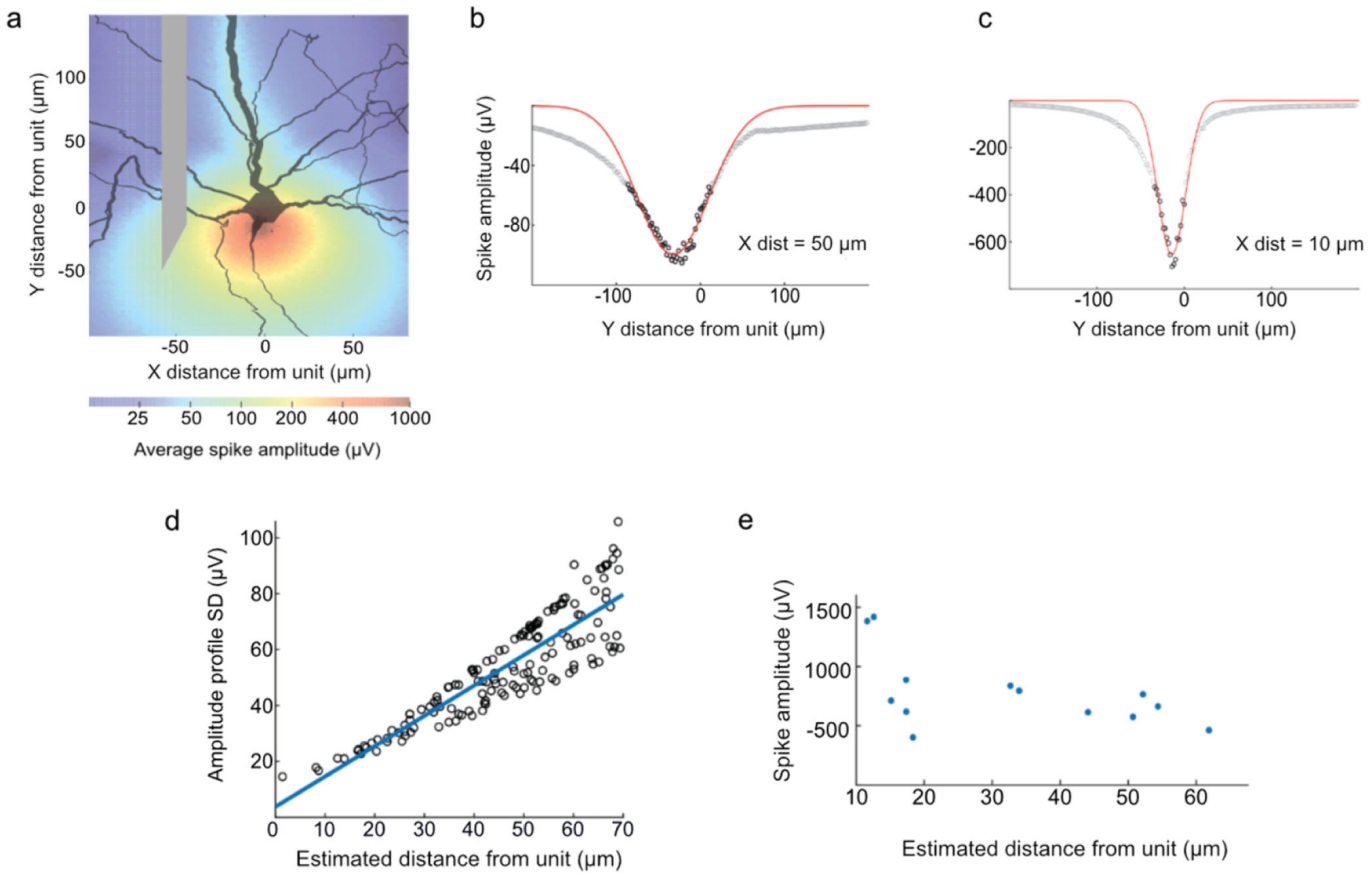
Estimation of the distance between the electrode and recorded neurons (a) Heatmap showing the simulated spike peak amplitudes at different positions around a mitral cell (b), (c) Modelled spike amplitudes (black circles) at different positions along the electrode axis, The electrode was moved across the *Y* axis, for different fixed distances X from the axon initial segment. Red lines show the Gaussian fit at 50 *µ*m (b) and 10 *µ*m (c) from the initial segment of the biophysically detailed model mitral cell. (d) Relationship of the standard deviation of fitted spike amplitudes profiles and the distance of the electrode axis to the initial segment of the neuron. (e) Estimated electrode distance from the mitral cell initial segment and the peak amplitudes of the maximal recorded action potentials, collected experimentally in the MOB.

To more precisely estimate the position of the jULIEs sensor relative to the recorded neurons, we calculated the electrical field around biophysically realistic models of mitral cells (figure 4(a)), and estimated the resulting spike amplitudes during axial movement of the electrode (figures 4(b) and (c)). Simulations of the extracellular electrical field demonstrated that the distance between the axis of electrode movement and the soma of the neuron could be predicted from the standard deviation of the amplitude profile (figure 4(d)). We thus performed further electrode insertions into the olfactory bulb while monitoring the change of spike amplitude during motion and estimated the distance from the recorded units (figure 4(e)). We found that stable recordings could be made at estimated (horizontal) distances as low as 10 *µ*m from the axon initial segment. This suggests that jULIEs, sharpened, electrochemically modified ultramicroelectrodes, could indeed approach and record near intact neurons.

### 3.5 Stimulation

**Figure 5:**
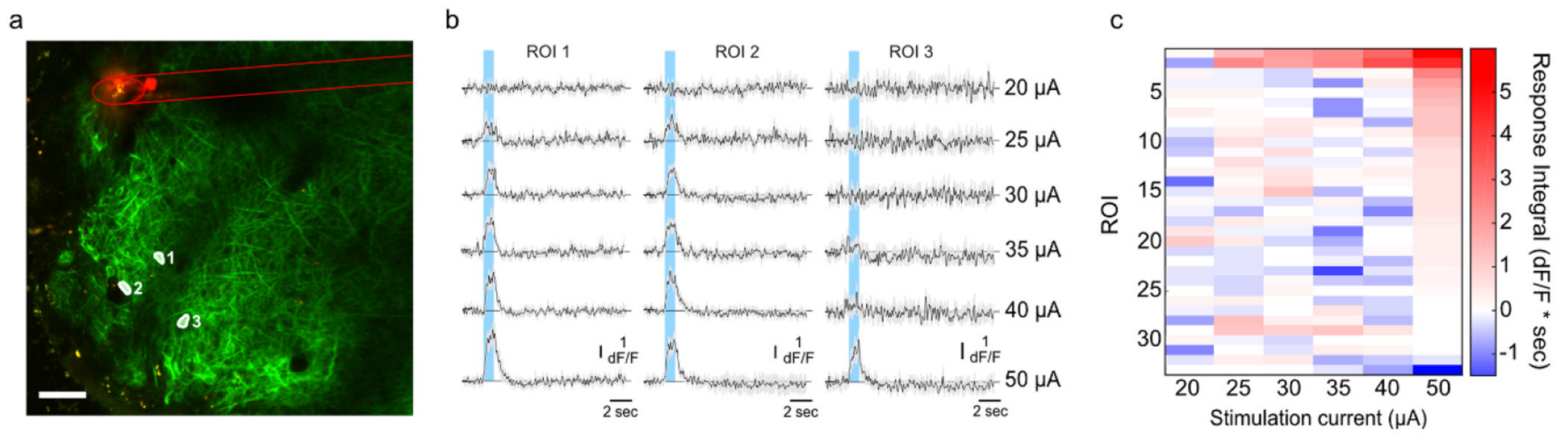
Electrical stimulation *in vivo* in the OB using a single jULIE probe (a) DiO labelled position of the jULIE stimulation site relative to ROIs labelled 1, 2, 3 in the OB of a mouse expressing the genetically encoded Ca^2+^ indicator GCaMP6f in projection neurons (Ybet-Cre x Ai95, see Methods, scale bar: 50 *µ*m). (b) responses of the 3 ROIs indicated in (a) to increasing stimulation current (stimulation period highlighted in blue). (c) The average response of all identified cells contained within the field of view (a) to increasing stimulation current.

A key advantage of the multi-step electrochemical modification described here is the large charge storage capacity due to the increased surface area and the stable reversibility of the Ir^3+^/Ir^4+^ redox couple [24, 45]. To assess whether this would indeed allow for electrical stimulation efficient enough to drive neuronal activity with ultramicroelectrodes, we performed microstimulation under 2-photon observation in the olfactory bulb of transgenic mice expressing the genetically encoded Ca^2+^ indicator GCaMP6f in projection neurons (Tbet-Cre x Ai95). Using a single jULIE fibre (figure 5(a)), 5–10, 1 ms long pulses were injected carrying currents from 2 to 150 *µ*A in 5 *µ*A increments (see Methods). Cells were found to respond to increasing stimulation levels, with most cells in the proximity of the stimulation site responding at 50 *µ*A injection current (figures 5(b) and (c)). Electrical properties of the interface remained stable throughout 200 000 injected voltage pulses (supplementary figure 6(a)). Moreover, EIS and CV characterization after current stimulation revealed only a three- to four-fold increase after 1 million pulses (supplementary figures 6(c) and (d)), enabling the use of the same probe for further recordings.

### 3.6 Customisable sensor layout

Siliconprobes are widely adopted because of their ability to perform extracellular recordings with relative ease on multiple channels. However, the underlying microfabrication methods have limitations in terms of materials, dimensions and required efforts to customize sensor layout to fit a certain target brain structure. In contrast, the layout of the recording sites of jULIEs can be readily customized (figures 1(e)–(i)) to best match the anatomical requirements of individual experiments. Microwires can be pre-arranged in various polytrode configurations, to maximize site lateral density using *µ*m-scale polymer templates (supplementary figure 1) which help splay fibres at insertion (supplementary video 2). To record from sensory areas in the mouse brain, 16-channel pre-arranged polytrodes were assembled: recording site layout was mapped onto the tonotopical arrangement in the auditory IC; or, as an evenly spaced one-layer polytrode arrangement, onto L5 neurons in the primary visual cortex.

### 3.7 Recordings in the inferior colliculus

**Figure 6:**
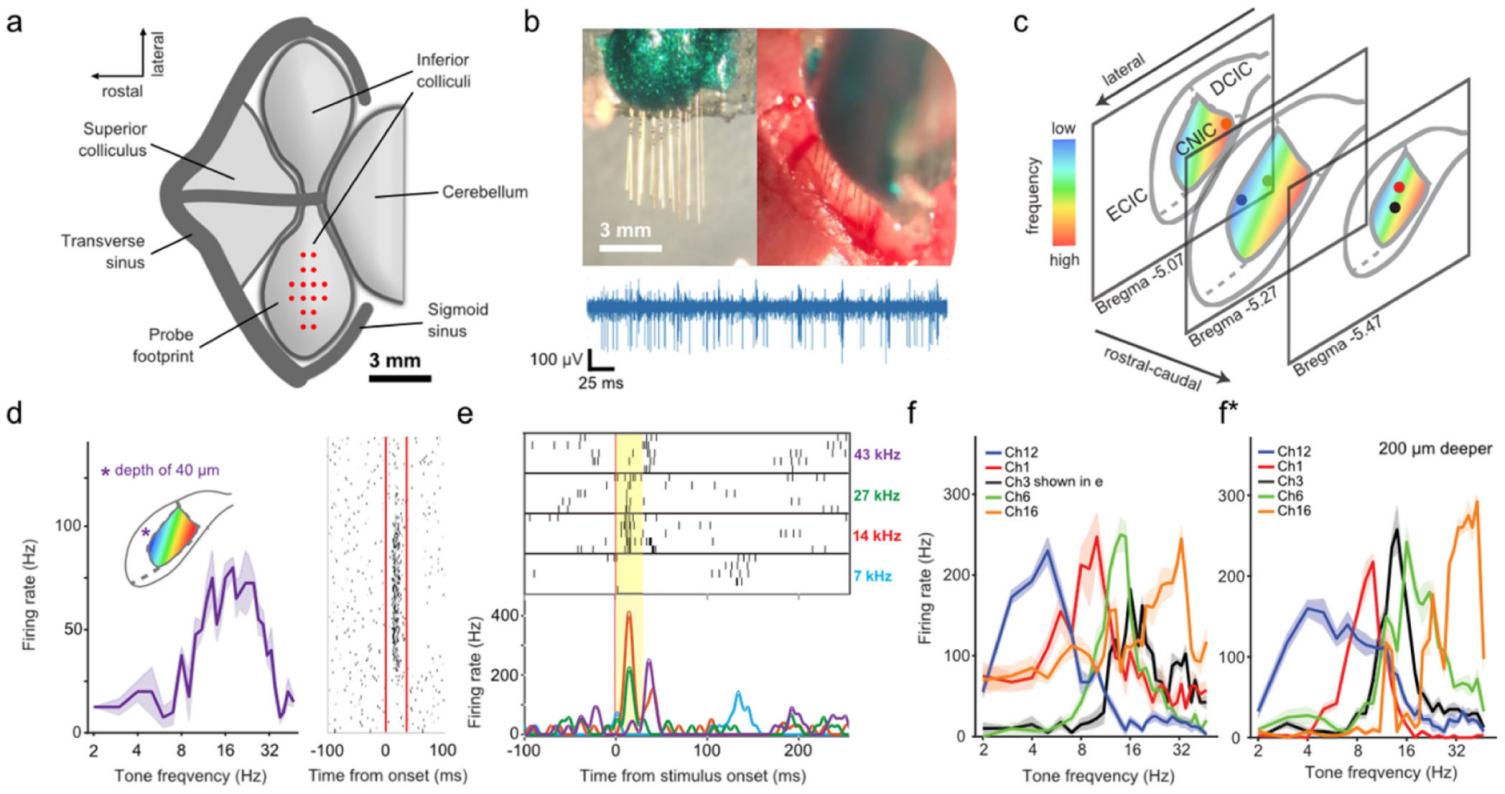
jULIE recordings in the inferior colliculus. (a) Dorsal view of the inferior colliculus (IC) as seen after craniotomy. (b, left) Top image of the 16-channel jULIE probe; (b, right), top view of probe entering the dorsal surface of the IC; (b, bottom), voltage trace with units recorded from IC. (c) Spatial distribution of recording sites used for the analysis in f and f ^∗^. Colours illustrate the arrangement of tonotopy in the IC, with low frequencies best represented in superficial/lateral regions and progressively higher frequencies in progressively deeper/more medial regions. The colour of the dots in (c) corresponds to the colour of the tuning curves shown in (f) and (f^∗^). (d) jULIE recording from a superficial location (~40 *µ*m below the dorsal surface; inset: schematic of recording location in IC dorsal cortex). Note the unimodal frequency tuning (left) and reliable, low-latency response (right); each dot represents a spike, red lines represent stimulus onset and offset respectively). (e) Evoked responses recorded from the same channel in the same location in response to 4 different tones (frequencies coloured shown on the left of the raster plot; red line: stimulus onset; stimulus duration: yellow box), each repeated five times. Different frequencies evoked different response patterns. While 14 and 27 kHz elicited short-latency responses, 7 kHz elicited no response and 43 kHz elicited a late response. Sparse spontaneous activity is seen before and after the sound presentation. (f) Tuning curves (average of activity evoked by 5 sound presentations) were recorded at different locations (colour-coded in c) illustrating position-specific preferred frequencies. Tuning curves are pronounced, unimodal, and discriminable between the units, recorded simultaneously from several different positions in the inferior colliculus. (f^∗^) Further inserting the jULIEs 200 *µ*m deeper into the IC maintained receptive field properties.

When sampling auditory physiology, it is often desirable to record along the tonotopic axis to resolve population activity evoked by different frequency components of an auditory stimulus. This often requires multi-shank probes which can be challenging in mice, as tissue damage around the multiple insertion sites is thought to limit the number of successfully recorded units [5, 18, 54]. Thus, to sample from the three-dimensional volume of the IC, a 4-layer stacked jULIE probe with a rectangular footprint (figure 6(a)) was assembled. Individual probe shanks (figure 6(b)) were separated horizontally by ~100 *µ*m, recording sites were vertically arranged at 100 *µ*m steps matching the slope of the isofrequency planes and inserted into the IC of anaesthetized mice with the deepest shank next to the medial axis (figure 6(c)). Unit activity in the IC was readily resolved (figure 6(b)) with presentation of pure tones. Notably, while recordings from superficial layers are generally difficult to obtain [55], jULIEs allowed to record evoked spiking activity with clear tuning in superficial layers of the IC already at ~40 *µ*m from the surface (figure 6(d)). Overall, the probe mapped onto the tonotopy of the IC (figures 6(e) and (f)) and recorded tone evoked responses across the two-dimensional plane (figures 6(f) and (f*)), including very superficial layers.

### 3.8 Recordings in the visual cortex

**Figure 7:**
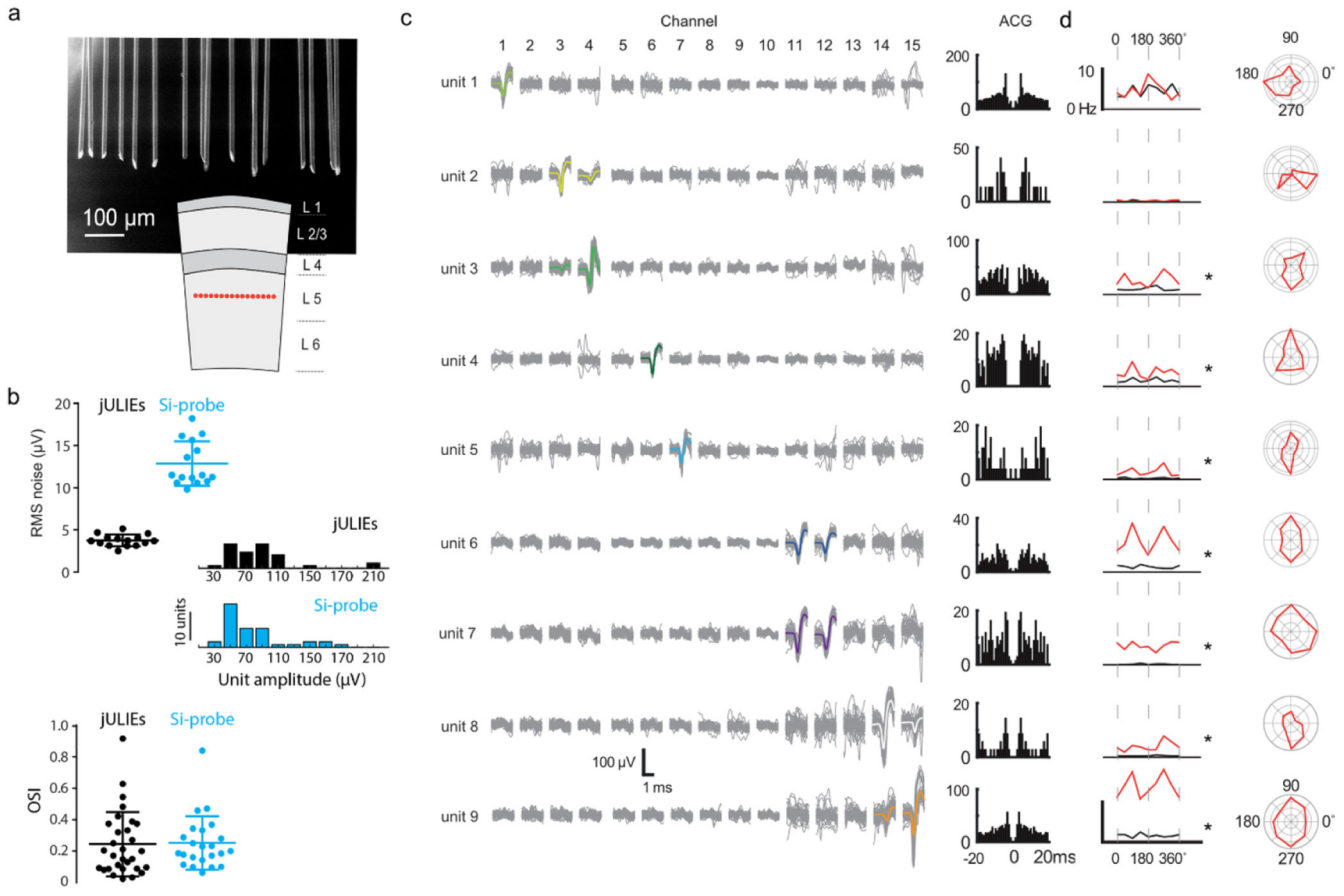
jULIE recordings in visual cortex. (a) SEM micrograph of the jULIEs recording site layout and their positioning in layer 5 (red dots in the bottom schematic). (b) jULIEs vs Si-probe: RMS noise distribution, histogram of average spike amplitudes (negative deflection) from single units and distribution of orientation selectivity index for all units recorded using jULIEs and Si-probe. (c) left: waveforms of all units separated during a single jULIE penetration (650 *µ*m depth). Average spike waveforms are shown in colour. Right: auto-correlograms (ACG) of spike times for separated units. (d) left: Visual responses for the units shown in (c) for eight different drifting directions (red) and baseline firing rate (black). Asterisks denote significant evoked activity in at least one direction. Right: polar plots showing normalized tuning curves.

The neocortex is a layered structure where targeting individual layers with recording sites for extracellular recordings in a laterally dense fashion is challenging, yet often desirable. While e.g. silicon-polytrodes [56] can achieve single layer multi-channel recordings in the cortex, their larger shank thickness and increased friction and tissue dragging result in local tissue damage. As a result, the necessary spacing of simultaneously inserted shanks limits the site density that can be achieved within the layer. jULIEs in turn can be arranged in arbitrary 2D and 3D patterns, such as a single-layer horizontal site layout (figure 7(a)), to target recording sites to individual layers. Due to the morphology and surface properties of individual shanks, recording sites can be spaced as close as 50 *µ*m with minimal tissue disturbance.

We tested the utility of the laterally dense probes by recording from the primary visual cortex (V1) of lightly anaesthetized mice at cortical depths corresponding to layer 5 (500–750 *µ*m down from pia surface). Following surgery to expose the cortex, a 16-channel probe with 50 *µ*m inter-shank spacing was lowered into the cortex. Separate recordings were performed under similar conditions with a comparable 16-channel Si-probe (four shanks 150 *µ*m apart, each containing 4 recording sites in a tetrode arrangement, see Methods). Several recording sessions, at least 50 *µ*m apart in-depth, were made both with the jULIEs and the Si-probe.

Recordings from individual jULIEs had lower noise (RMS 3.77 ± 0.69 *µ*V; *n* = 15 wires) compared to the Si-probe used in this experiment (RMS 12.88 ± 2.64 *µ*V; *n* = 15 sites, figure 7(b)). Single units appeared on multiple wires (e.g. unit 7, figure 7(b)), and the same wire could record from multiple single units (e.g. ch15, figure 7(b)). Average spike amplitudes of well-separated units showed a comparable distribution to those from the Si-probe (figure 7(d), jULIEs: 66.39 ± 45.48 *µ*V, *n* = 33; Siprobe: 77.44 ± 40.40 *µ*V, *n* = 39; *p* = 0.28, Student’s t-test). Recording quality was comparable and on many channels exceeding Silicon probes. Meanwhile, with a shank spacing of 50 *µ*m, recording sites were laterally substantially denser than possible with vertical Si-probes.

Well-isolated single units (see Methods for sorting procedures) could be detected on single or multiple (usually 2) neighbouring recording sites (figure 7(b)). We recorded visually evoked single-unit activity using standard drifting gratings (eight directions, see Methods for details). As expected [57], activity evoked by visual stimuli showed characteristic tuning curves (figure 7(c, right)) for single units. Units recorded on the same channel (e.g. units 6 and 7) had different levels of evoked activity and markedly different tuning, consistent with the assumption that units represent individual neurons. Furthermore, the single units responsive to drifting-gratings recorded from jULIEs and the Si-probe had a similar distribution of orientation selectivity index (jULIEs OSI: 0.24 ± 0.20, *n* = 30 units; Si-probe OSI: 0.25 ± 0.17, *n* = 23 units; *p* = 0.88, Student’s t-test, figure 7(d)).

**Figure 8:**
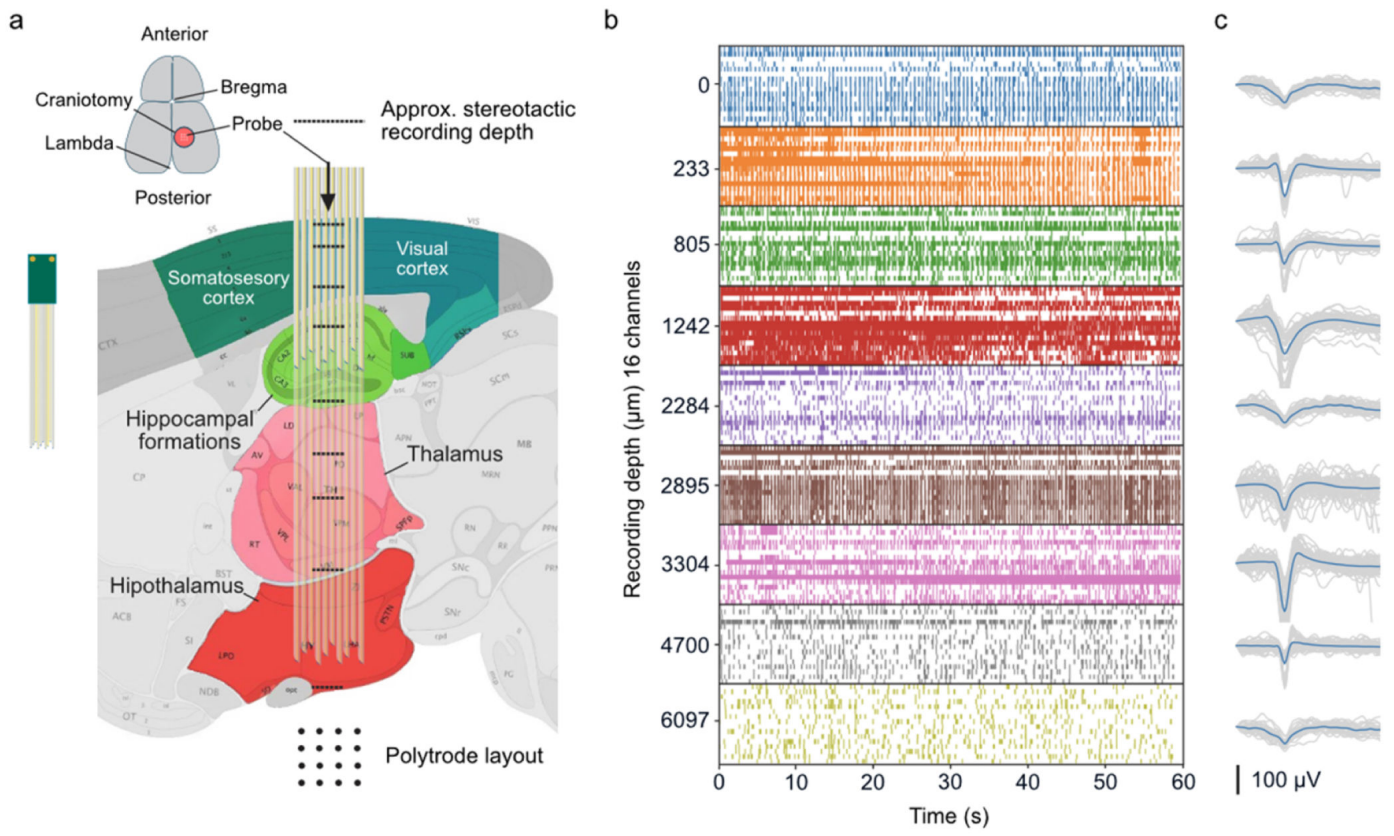
jULIE recordings from deep brain areas. (a) Schematic (sagittal section) of vertical array insertion route and static recording positions throughout the depth of mouse brain. (b) Recordings of spontaneous neural activity at insertion—raster lines represent individual channels at each depth. (c) Waveforms of units were detected across 16 channels at depths shown in (b).

Finally, to assess whether jULIEs are amenable to recordings deep in the brain we employed a 16 channel polytrode and inserted it through the visual and somatosensory cortex, hippocampus, thalamus down to the hypothalamus (figure 8). Stable units were recorded in any of these structures down to >6 mm below the cortical surface.

## 4 Discussion

Here we have described the manufacturing, modification and use of ‘jULIEs’, glass-metal composite ultramicroelectrodes, as a minimally invasive and scalable approach to record high-quality extracellular signals from the intact mouse brain. We have demonstrated that multi-step modifications with nanomaterials substantially reduced interfacial impedances, decoupling wire dimensions and materials from the performance of the interface to respond to the biological signal. jULIEs were compliant with the tissue structure, causing no detectable nuclear displacement or damage to the blood-brain-barrier. While the current study focuses on acute recordings, this makes them an ideal candidate for long-term implantation, a key goal for future studies. The minor local tissue disturbance was likely due to the overall small dimension of individual fibres [5, 6], cylindrical form, sharpened tip and the smooth shanks of individual fibres (figures 1(j)–(l)) which have been suggested to keep mechanical interaction with surrounding tissue minimal [38]. Other advantages of modified microwires are their composite nature: metal cores are effectively fused with the glass ensheathing providing a mechanically robust, defect- and delamination-free very low parasitic-capacitance insulation [21]. Polymer coated microwires [13], microfabricated polymer electrodes [8, 58, 59], carbon fibres [6, 7], multimodal pipettes [60], syringe-injectable mesh electrodes [61, 62], niobium microwires [63], amorphous SiC [64] and active-CMOS probes [65] are promising avenues for potentially low-damage neural recordings. Several approaches have been put forward to allow for their connectoriztion to standard readout electronics however at large scale (above 10k) these remains a challenge. Due to their metal core, the jULIEs can be readily connected to integrated read-out electronics in a scalable manner, through direct bonding on a smaller scale (supplementary figure 7) or through non-thermal flip-chip bonding at very large scale [21, 39]. Moreover, when assembled into customized neural probes, the overall layout of sensor sites can be individually tailored to fit the neuroanatomical structure in three dimensions.

Multi-step electrochemistry enables decoupling of the recording interface in contact with the neuronal tissue from the material properties of the wires and their limitations. This may allow usage of core compositions with further improved wetting capabilities which are needed to manufacture thinner fibres. NanoAu deposition could then restore the contact surface area and electrochemical compatibility, and, in tandem with IrOx deposition, provide a low impedance electrode–electrolyte interface despite sub-micrometre core diameters. In fact, microwire modification with an inert metal such as the nanoAu enables access to a diverse set of impedance reducing strategies with materials such as polymers, carbonaceous or hybrid materials. Furthermore, nanoAu or functionalisation with other inert metals are compatible with a wide range of possible biofunctionalization strategies, to detect e.g. pH [66], catecholamines [67], or genetic material [68]. Finally, through multi-step electrochemistry we have created high rugosity surfaces in combination with a reversible redox couple at the electrode-tissue interface, resulting in an attractive and stable substrate for microstimulation purposes.

The limited tissue disturbance allowed us to record single units on several adjacent jULIEs (figure 3(g)), even when the electrodes were axially inserted and retracted multiple times (figure 3(f)). In combination with the flexibility of geometric custom arrangement of recording sites, this could be used to tailor probe arrays for optimal unit isolation, maximizing unit yield.

For understanding the brain, recording and stimulation in three dimensions at high speed, and at a large, distributed scale is critical. Various imaging, electrical or genetic approaches are available, each challenged by particular limitations of scalability [69]. Currently, the technologies that not only might be closest to that goal but are also applicable to clinical challenges are electrical recording and stimulation techniques. Unlike planar probes, which are limited to a distribution of sites along a vertical axis, jULIEs probes, due to the minimal shank size, can be shaped to cover any distribution of sites. This can be highly advantageous in many experimental situations as demonstrated by the various recording use-cases here. While modern silicon probes are considered minimally disturbing to the tissue, and multiple shank recordings are possible, their cross-sectional area (e.g. 1680 *µ*m^2^ for the cutting-edge Neuropixel 2.0 probes) is still significantly larger than 20 *µ*m microwires (314 *µ*m^2^ per wire), which means that when recording from planarly distributed cell populations, the jULIEs probes have advantages. We consider the two approaches complementary, which should be considered in every recording scenario.

Here, we introduce jULIE probes that combine low invasiveness with high-quality electrical recording and stimulation, thus offering a platform for interfacing with brain activity at brain-wide scale with single-cell resolution.

## Supplementary Material

supplementary figure 1

supplementary video 1

supplementary video 2

## Data Availability

The data generated and/or analysed during the current study are not publicly available for legal/ethical reasons but are available from the corresponding author on reasonable request.
