# Supplementary material for: jULIEs: nanostructured polytrodes for low traumatic extracellular recordings and stimulation in the mammalian brain
Source: J Neural Eng. Author manuscript; Available in PMC 2025 Oct 21. (PMC7618266; doi:10.1088/1741-2552/ac514f)
Supplement: supplementary figure 1 [file EMS209499-supplement-supplementary_figure_1.pdf]

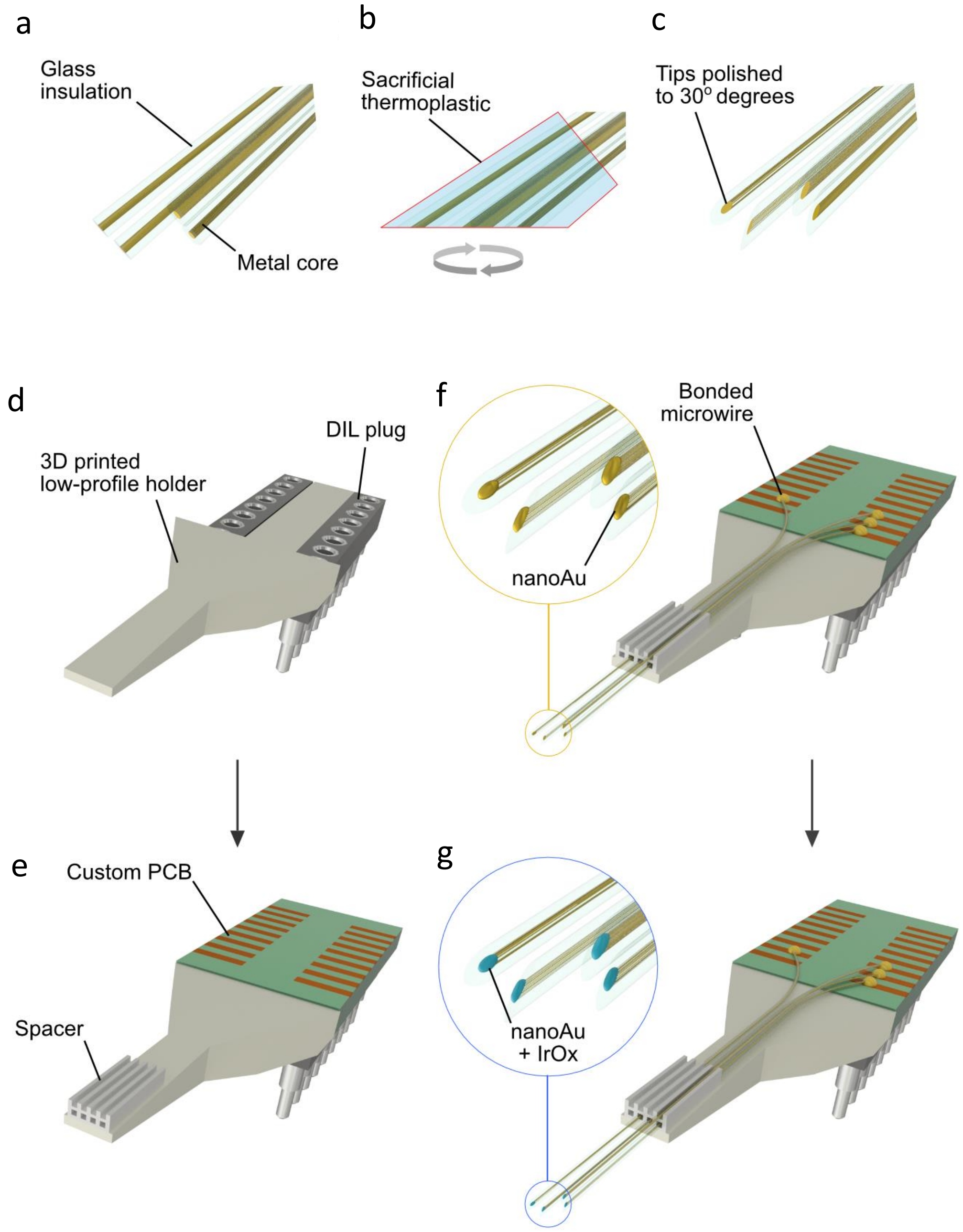

Supplementary Figure 1. Preparation and assembly of jULIE probes. **(a)** Microwires were grouped into bundles containing several thousand wires and embedded into Crystalbond 509. **(b)** Samples were fixed into a holder and polished on a Buehler 250 using 5 grits. **(c)** Sharpened wires were de-embedded overnight by solubilization in Crystalbond Stripper. **(d,e)** Low profile microwire holders, a custom PCB and connector plug were assembled. **(e)** Wire spacers were placed at the tip of the 3D printed holder. **(f)** Microwires were inserted into spacers and built-up layer by layer. **(g)** Microwires were bonded and modified with nanoAu and IrOx and characterized by EIS.

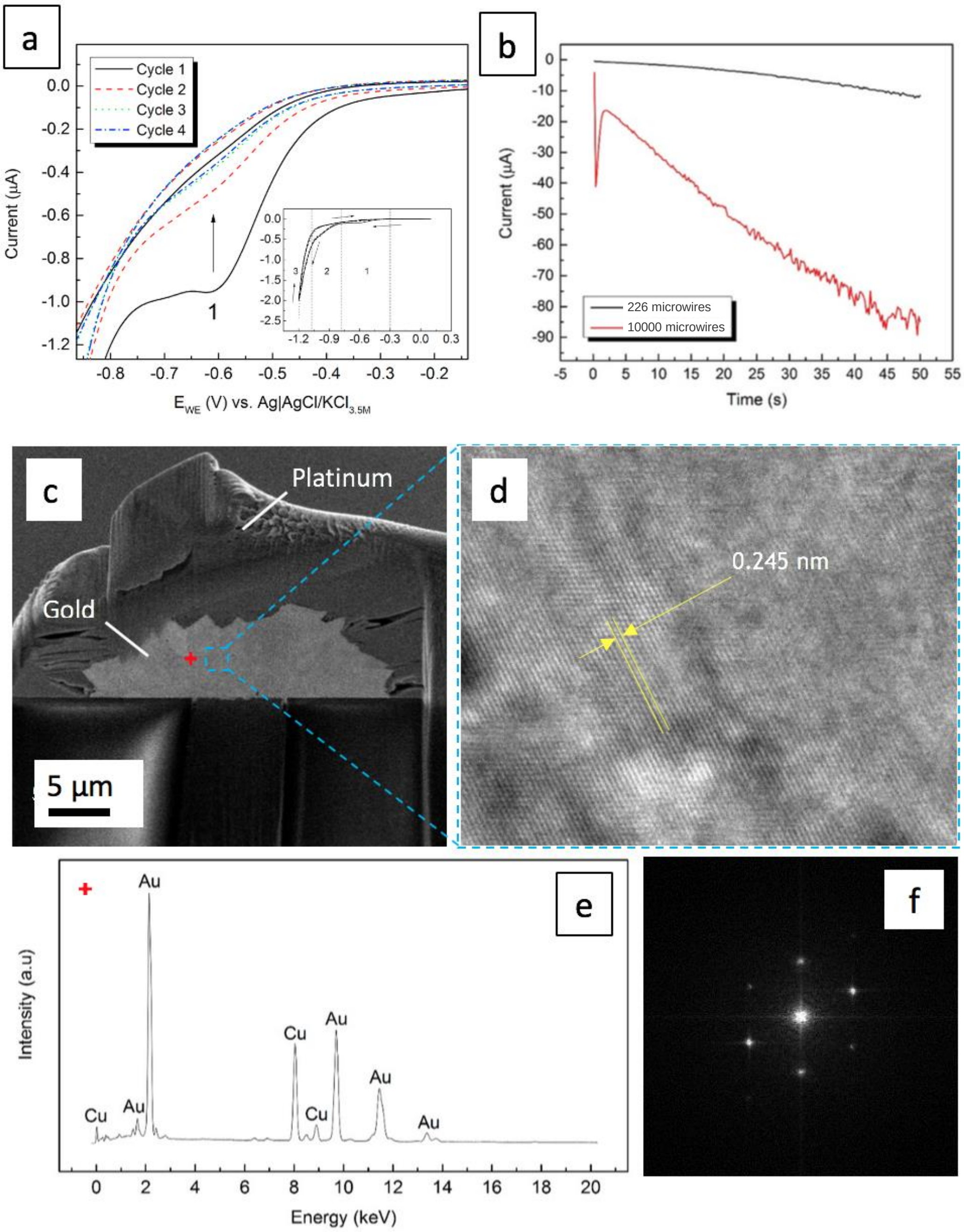

Supplementary Figure 2. Two-step nanoAu electrodeposition on polished gold/glass ultramicroelectrodes from an additive free cyanide electrolyte. **(a)** Peak current (1) response for nanoAu electrodeposition during first voltammetry cycle at 50mVs^{-1} between -1.2V and 0.2V vs. REF. **(b)** Potentiostatic plating of nanoAu on bundles containing several tens of individual glass-gold jULIEs. **(c)** FIB-SEM processed electron-transparent slice from the nanoAu deposit. Note the protective Pt layer deposited to facilitate FIB milling. **(d)** STEM image of Au atomic lattice arrangement. **(e)** EDS spectra of point marked by a red cross in (c). **(f)** FFT X-ray diffraction pattern indicating the single crystal nature of sample from panel (d)

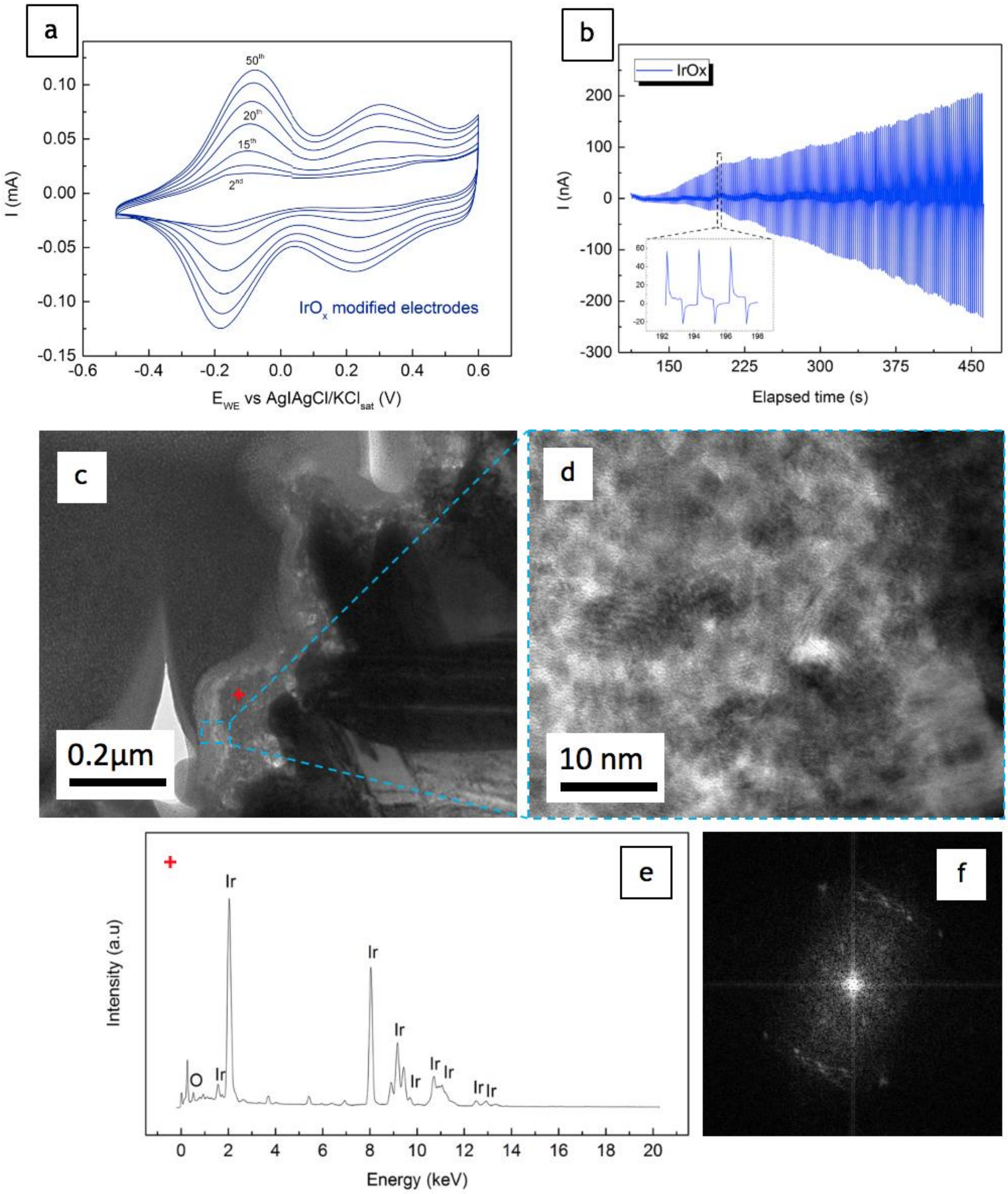

Supplementary figure 3. Two-step IrOx electrodeposition on nanoAu. **(a)** Cyclic voltammetry electroplating on nanoAu at a sweep rate of $50 mVs^{-1}$ between -0.5V and 0.6V vs. REF. **(b)** Potentiostatic IrOx pulse-plating on nanoAu. **(c)** STEM micrograph of the electrodeposited IrO_x thin-film. **(d)** STEM of bulk IrO_x thin-film showing polycrystalline aggregates irregularly packed resulting in the porous overall structure. **(e)** Energy dispersive x-ray diffraction spectra of the IrO_x thin-film at position indicated by a red cross in (c). **(f)** FFT X-ray diffraction of polycrystalline IrO_x showing typical polycrystalline response pattern.

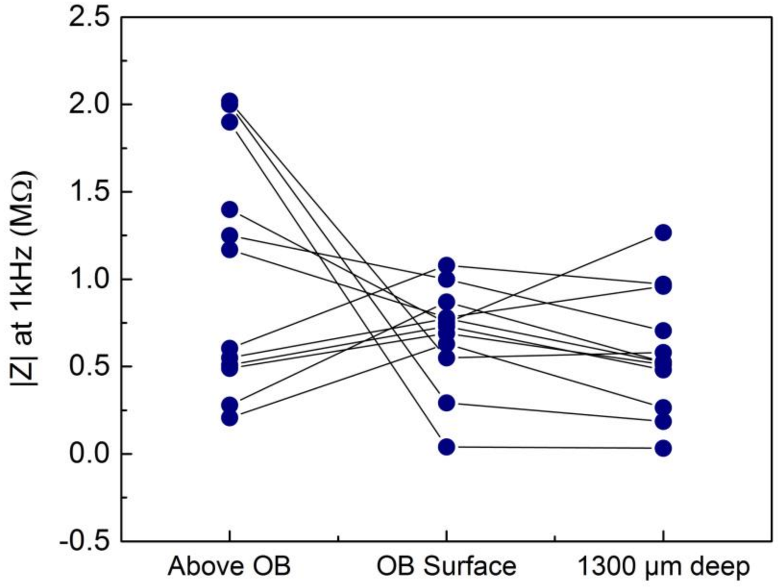

Supplementary figure 4. Impedance stability of the jULIE probes at different stages during an *in-vivo* experiment in the mouse olfactory bulb: before insertion in ACSF ("Above OB"), touching the surface of the OB (dura removed) and after insertion to ~1300 µm into the olfactory bulb.

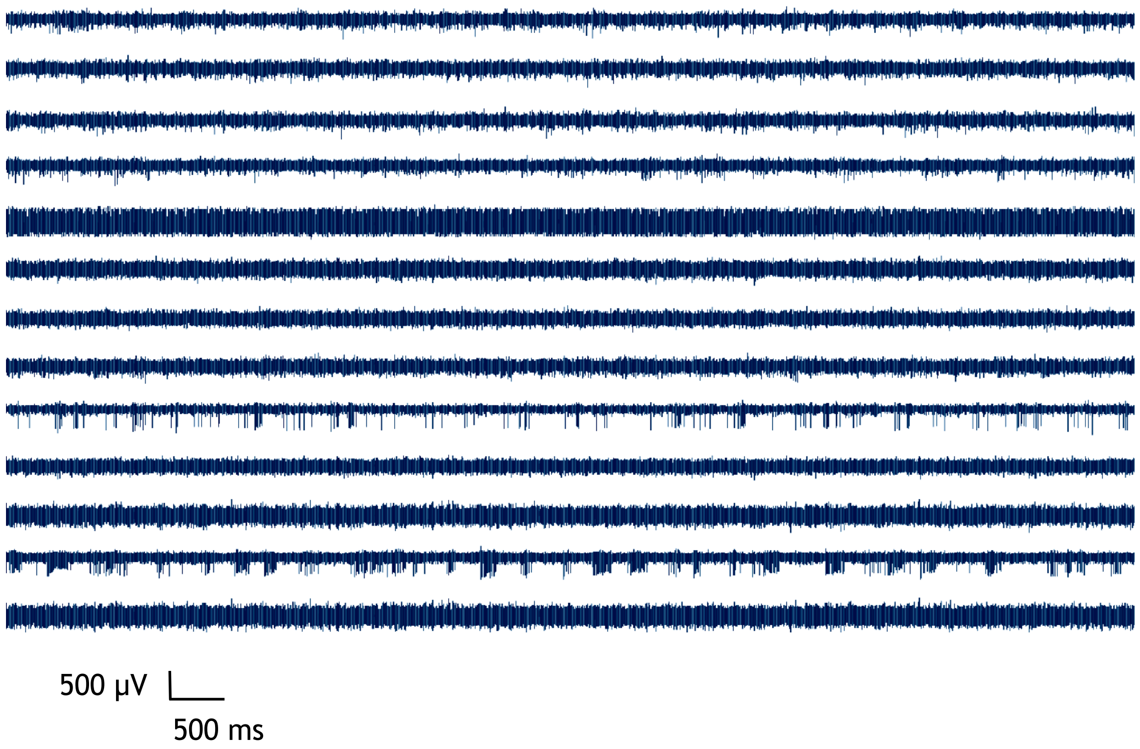

Supplementary figure 5. *In-vivo* extracellular recordings at ~400 µm deep in the main olfactory bulb of an anesthetized C57BL/6 mouse using a 13 channel jULIE polytrode.

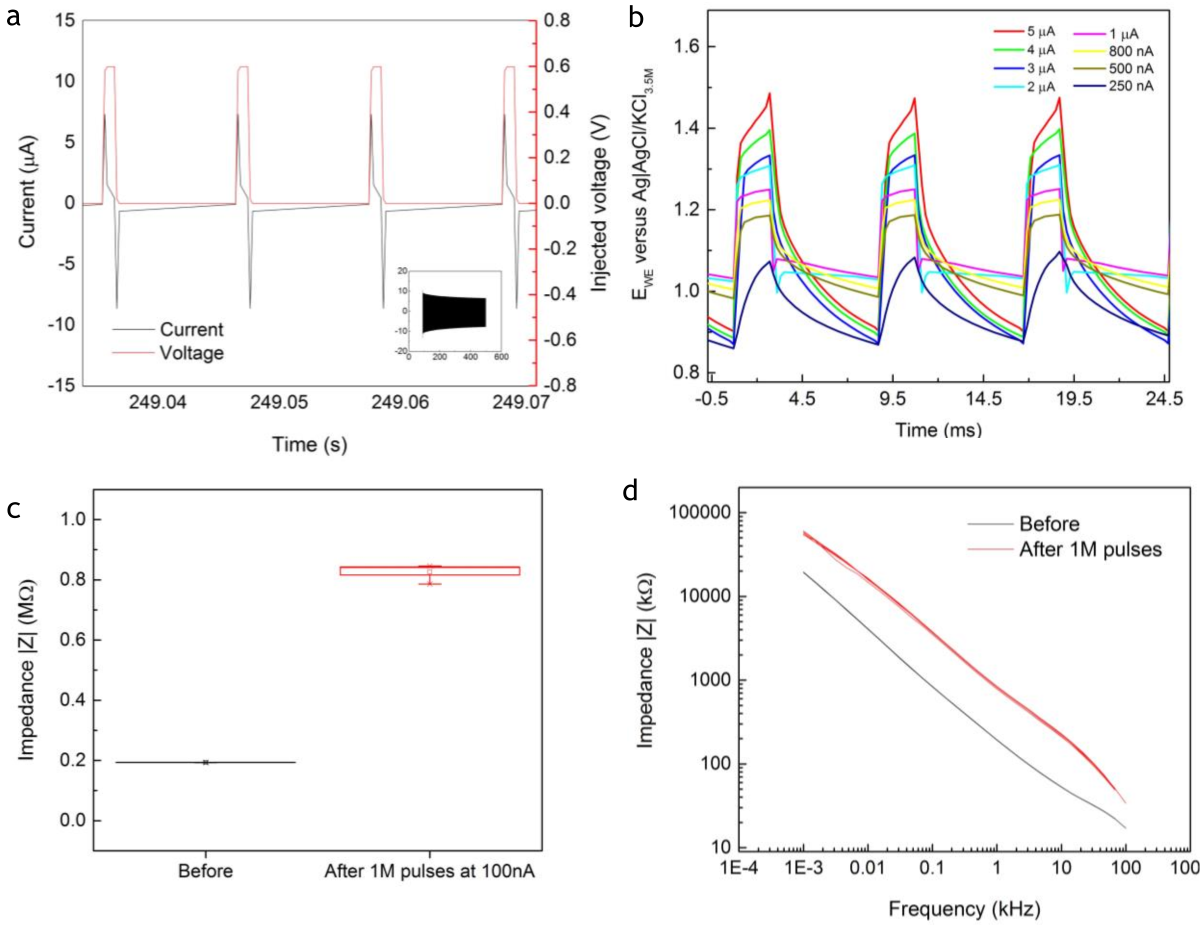

Supplementary figure 6. (a) Characterization of voltage stimulation performance of nanoAu + IrOx in cortex buffer for injected 1 ms long 600 mV square pulses. **(b)** Electrode voltage response to different injected currents. **(c)** Impedance response after 1M 100nA injected pulses and overall EIS response **(d)**.

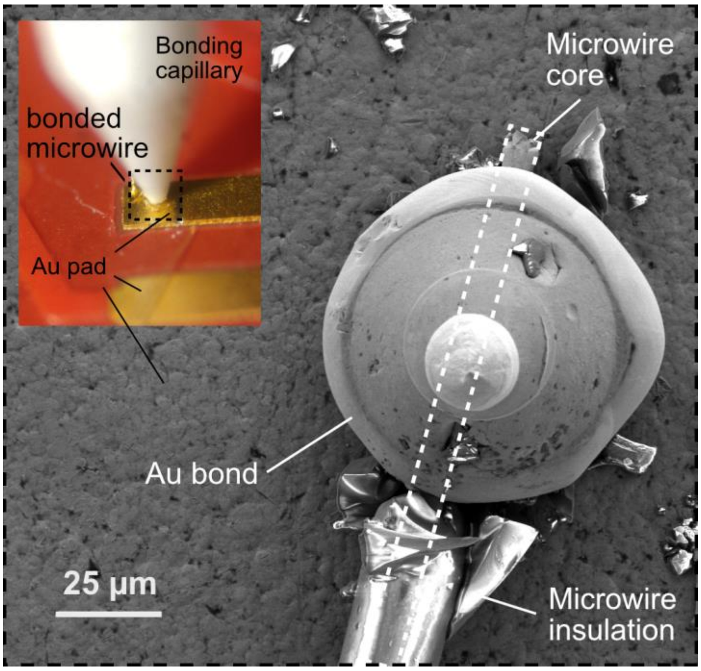

Supplementary figure 7. jULIE wire core bonded to gold pad after removal of glass insulation. Dry contact resistances range between 1Ω and 8Ω

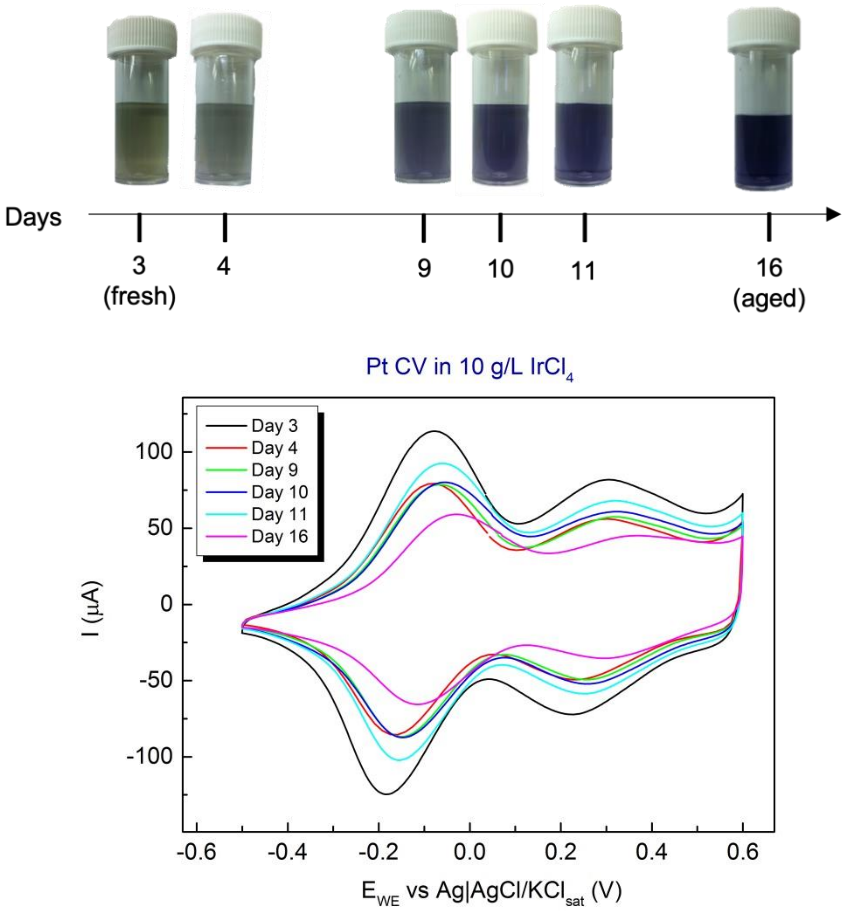

Supplementary figure 8. After preparation the electrolyte was 'aged' in standard laboratory conditions. Specifically, a change in the bulk color from yellow to dark blue was recorded as shown in the top plot. By cyclic voltammetry the change in the redox response over the ageing process was recorded. Dark blue at day 16 is considered stable to yield desired IrOx films described in Methods section and characterized in Supplementary figures 2 and 3.
